# *Operando* monitoring of a room temperature nanocomposite methanol sensor†

**DOI:** 10.1039/d2cy01395a

**Published:** 2022-12-14

**Authors:** Qaisar Maqbool, Nevzat Yigit, Michael Stöger-Pollach, Maria Letizia Ruello, Francesca Tittarelli, Günther Rupprechter

**Affiliations:** a Department of Materials, Environmental Sciences and Urban Planning (SIMAU), Università Politecnica delle Marche INSTM Research Unit, via Brecce Bianche 12 60131 Ancona Italy; b Institute of Materials Chemistry TU Wien, Getreidemarkt 9/BC A-1060 Vienna Austria guenther.rupprechter@tuwien.ac.at; c University Service Center for Transmission Electron Microscopy TU Wien, Wiedner Hauptstr. 8-10 1040 Vienna Austria

## Abstract

The sensing of volatile organic compounds by composites containing metal oxide semiconductors is typically explained *via* adsorption–desorption and surface electrochemical reactions changing the sensor's resistance. The analysis of molecular processes on chemiresistive gas sensors is often based on indirect evidence, whereas *in situ* or *operando* studies monitoring the gas/surface interactions enable a direct insight. Here we report a cross-disciplinary approach employing spectroscopy of working sensors to investigate room temperature methanol detection, contrasting well-characterized nanocomposite (TiO_2_@rGO-NC) and reduced-graphene oxide (rGO) sensors. Methanol interactions with the sensors were examined by (quasi) *operando*-DRIFTS and *in situ*-ATR-FTIR spectroscopy, the first paralleled by simultaneous measurements of resistance. The sensing mechanism was also studied by mass spectroscopy (MS), revealing the surface electrochemical reactions. The *operando* and *in situ* spectroscopy techniques demonstrated that the sensing mechanism on the nanocomposite relies on the combined effect of methanol reversible physisorption and irreversible chemisorption, sensor modification over time, and electron/O_2_ depletion–restoration due to a surface electrochemical reaction forming CO_2_ and H_2_O.

## Abstract 2

A cross-disciplinary approach is employed to study working nanocomposite room temperature methanol sensors *via* a combination of operando and *in situ* spectroscopy techniques, paralleled by resistance measurements.

## Graphical abstract

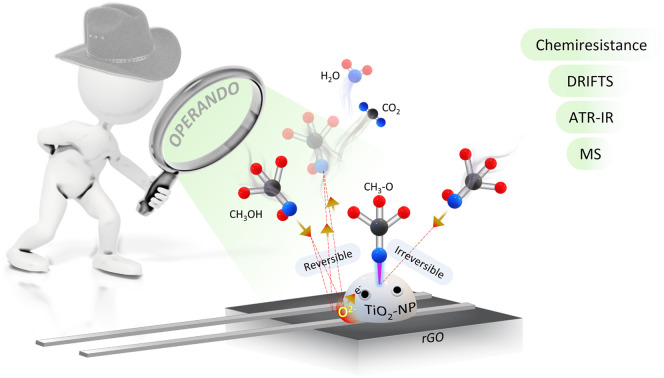

## Introduction

Methanol, a volatile organic compound (VOC), is extensively used as an organic solvent in chemical, biomedical, pharmaceutical and other industries.^1,2^ It may also serve as a cost-efficient and clean-burning liquid fuel for automobiles, limiting emissions of sulphur oxides (SOx), nitrogen oxides (NOx) and particulate matter to the environment.^3^ However, methanol is toxic as it can be absorbed through the skin or lungs leading to methanol poisoning. Moreover, methanol toxicity increases *via* metabolism to formaldehyde and formic acid, which upon accumulation leads to adverse health effects.^4,5^ This illustrates the importance of an efficient methanol sensor capable of detection in an ambient environment.

Metal oxide semiconductors (MOScs) such as In_2_O_3_, ZnO, WO_3_, SnO_2_, NiO, Co_3_O_4_, CuO, *etc.* have been widely used as VOC gas sensing materials, based on their resistivity change upon exposure to analytes (chemiresistivity).^6–12^ To achieve higher sensitivity at relatively low working temperatures, MOScs have been doped with noble metals such as Au, Ag, Pt, Pd, *etc.*^13–16^ Still, conventional MOScs typically require working temperatures of 90–400 °C to desorb water chemisorbed on the sensor surface and to provide free sites for analyte chemisorption.^17^ High MOSc working temperatures maintain the sensitivity toward VOCs over repeated cycles,^18,19^ but induce high-power consumption, reduce the sensor lifetime and pose a security risk for sensor operation in a flammable environment.^6–11^

The next generation of VOC sensors thus operates at room temperature (RT), benefitting from power-saving, eco-friendliness, sustainability, and higher safety.^20,21^ Recently, several materials have been tested as RT-VOC sensors, such as carbon-based materials including multiwall carbon nanotubes with polyaniline, carbon derivatives in polyetherimide with a liquid crystal polymer, and graphene doped polyaniline. These sensors were able to detect methanol vapor of 50–500 ppm, 300–1200 ppm, and 50–100 ppm, respectively.^22–24^ Moreover, MOSc based materials, such as CdO with polyaniline and a thin layer of Au, Ag, Pt or Cu on top of In_2_O_3_–SnO_2_, have also shown detection limits of 100 ppm and 200–900 ppm, respectively. When In_2_O_3_ was combined with Ti_3_C_2_T_*x*_ (an MXene), methanol detection was further enhanced (5–100 ppm). Nevertheless, CdO and In_2_O_3_ possess significant toxicity to humans: carcinogenic effect, renal toxicity, alveolar proteinosis, emphysema or interstitial fibrosis.^25,26^ Thus, in terms of biocompatibility, using TiO_2_ nanomaterials as RT-VOC sensors has attracted attention. TiO_2_ nanotubes and quantum dots exhibited promising methanol sensitivity of 100–300 ppm and 1000 ppm at RT, respectively^27,28^ However, the sensors' response time and detection limit were rather high or required UV light for sufficient performance.

For MOSc-based VOC sensing devices, changes in electrical resistance and response–recovery times upon exposure to variable VOC concentrations have been reported repeatedly, but the sensing mechanism was often described rather hypothetically.^17^ To obtain a fundamental understanding of the sensor's functional properties rather requires real-time studies of the working sensor surface interacting with VOCs, *i.e.*, an *in situ* or *operando* approach hardly attempted before. Even more so as VOCs may irreversibly bind to MOScs, limiting the sensor functionality. For instance, methanol irreversibly adsorbs on nanocrystalline TiO_2_ and requires ≈425 °C for removal of the adsorbed methoxy species.^29^ Similarly, methanol adsorbs dissociatively on pure ZnO, as well as on ZnO decorated with Au nanoparticles, thus requiring 275–320 °C for operation. It was also reported that Au enhanced the ZnO reducibility and facilitated the generation of oxygen vacancies,^30^ which is considered an important factor in VOC sensing by MOSc surfaces.

To improve the current understanding, we have combined and linked surface sensitive vibrational studies^31–33^ of metal/oxide nanomaterials with RT sensor technology, simultaneously monitoring methanol–surface interactions and the corresponding resistance changes in real time. *Operando* diffuse reflectance infrared Fourier transform spectroscopy (DRIFTS) and *in situ* attenuated total reflectance-Fourier transform infrared spectroscopy (ATR-FTIR) were applied to evaluate the methanol gas sensing performance of green-synthesized and well-characterized TiO_2_ nanoparticles, reduced graphene oxide (rGO) and their nanocomposite (TiO_2_@rGO-NC). Based on simultaneous mass spectroscopy, the mechanism of room temperature methanol gas sensing was directly confirmed. Furthermore, the multidisciplinary approach presented herein may stimulate analogous in-depth studies and the development of versatile RT-VOC sensors.

## Experimental

### Materials

Titanium(iv) oxysulfate–sulfuric acid hydrate (TiOSO_4_·*x*H_2_SO_4_·*y*H_2_O) (Sigma-Aldrich®), graphite powder (Sigma-Aldrich®), methanol (analytical grade, by Sigma-Aldrich®), iso-propanol (Sigma-Aldrich®), potassium permanganate (>99%, Sigma-Aldrich®), l-ascorbic acid (99%, Sigma-Aldrich®), NaOH (Sigma-Aldrich®), deionized water (diH_2_O) (Milli-Q®), hydrochloric acid solution (37%, Merck®), olive leaf waste (kindly provided by Dr. Chiara Giosue, research fellow at Università Politecnica delle Marche, Italy).

A transparent glass substrate (dimensions: 3.2 cm × 2.4 cm), silver electrodes (purity = 99%, dimensions: 6 cm × 0.2 cm), precision digital multimeter (UNI-T® UT61E), sensor/electrode multimeter-connector (PalmSens®), hotplate magnetic stirrer-RH basic (IKA®, Germany), water bath ultrasonicator (FALC®, Italy), heating oven (LAPORTA®, Italy), electric grinder (KENWOOD® 500 W), centrifuge (ECCL31R Multispeed, Thermo Scientific®, USA), Brunauer–Emmett–Teller (BET) analyzer by Micromeritics®-ASAP-2020, X-ray powder diffractometer (Bruker®), TGA (Mettler®-851), UV/vis spectrometer (Lambda 750, PerkinElmer®), diffuse reflectance infrared Fourier transform spectroscopy (DRIFTS) using a Bruker Vertex 70 spectrometer with a DRIFTS cell (Pike), attenuated total reflectance-Fourier transform infrared (ATR-FTIR) spectrometer (Vertex-70, Bruker Optics®), ZnSe crystal (dimensions: 52 mm × 20 mm × 2 mm), quadrupole mass spectrometer (QMS) (Prisma Plus QMG 220, Pfeiffer Vacuum), high resolution transmission electron microscopy (HR-TEM) using an FEI TECNAI F20 field emission microscope.

### Synthesis and characterization procedures

#### Synthesis of the sensor materials

**Fig. 1:**
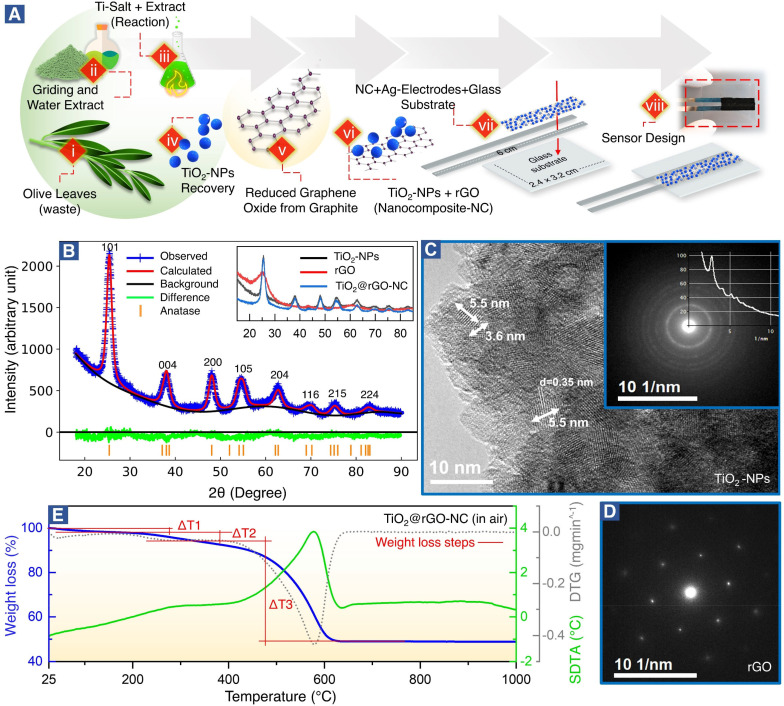
(A) Schematics (steps i to viii) elaborating the green synthesis of titanium oxide nanoparticles (TiO_2_-NPs), a nanocomposite (NC) of TiO_2_-NPs and reduced graphene oxide (TiO_2_@rGO-NC), and the sensor design (see Experimental for more details). (B) Crystal structure of TiO_2_-nanoparticles (TiO_2_-NPs) *via* Rietveld refinement. Inset: Measured diffractograms of the as-synthesized TiO_2_-NPs, reduced-graphene oxide (rGO) and nanocomposite (TiO_2_@rGO-NC). (C) HR-TEM image of unsupported TiO_2_-NPs, inset: SAED pattern of TiO_2_-NPs. (D) SAED pattern of rGO. (E) TG analysis (blue), DTG analysis (grey) and SDTA (green) of TiO_2_@rGO-NC in air.

Green synthesis of titanium oxide nanoparticles (TiO_2_-NPs) and reduced graphene oxide (rGO) was achieved following a previously reported, but modified, method.^34,35^ Briefly, the green synthesis of TiO_2_-NPs and a nanocomposite of TiO_2_-NPs and rGO (TiO_2_@rGO-NC) and the sensor production were accomplished by the following eight steps shown in Fig. 1.

i. Mature olive leaves, as a potential waste harvested in November, were thoroughly washed with dH_2_O to remove solid particles (impurities) and dried at room temperature in the dark to avoid photodissociation of secondary metabolites. To ensure repeatability and to match the metabolic profile necessary for TiO_2_-NP synthesis, it is important that the olive leaves are collected at a specific time of the year (preferentially November). The harvested leaves should be stored at >57% humidity at room temperature for further use.^36^

ii. The dried olive leaves were ground to a fine powder. For water extract preparation, 25 g of the olive leaf powder was soaked in 500 mL of diH_2_O for 12 h, and then heated to 85 °C for 3 h in a hot water bath. The suspension was allowed to cool to room temperature and was filtered using Whatman filter paper no. 1 to obtain an olive leaf extract.

iii. 500 mL of the prepared extract in a reaction flask was put on a hot plate magnetic stirrer at 85 °C and 150 rpm. Thereafter, 0.1 M TiOSO_4_·*x*H_2_SO_4_·*y*H_2_O was added to the preheated extract. After 20 min, the pH of the reaction mixture was adjusted to ∼4 by dropwise addition of 3 M NaOH solution. Next, heating was turned off and the reaction mixture was cooled to room temperature while stirring at 150 rpm.

iv. TiO_2_-NPs were collected by centrifugation at 8000 rpm and washed three times with diH_2_O to remove uncoordinated secondary metabolites from the extract. TiO_2_-NPs were dried in a hot air oven at 90 °C for 24 h. Next, the dried TiO_2_-NPs were calcined at 350 °C for 3 h in a furnace before being kept in an airtight jar for further use.

v. The production of reduced graphene oxide (rGO) was achieved by adding 2 g of graphite powder in a reaction flask immersed in an ice-water bath and maintained below 10 °C. After this, 100 mL of concentrated H_2_SO_4_ was added under continuous stirring. Then, 6 g of KMnO_4_ was gradually added. Next, the suspension was stirred at room temperature for 20 min followed by 10 min ultrasonication and stirring (process repeated 10 times). Subsequently, the reaction was quenched by addition of 400 mL diH_2_O and further ultrasonicated for 2 h. The suspension pH was adjusted at ∼6 by addition of 1 M NaOH solution, which was further ultrasonicated for 1 h. Then, 20 g of l-ascorbic acid was dissolved in 200 mL diH_2_O and slowly added to the suspension at room temperature while increasing the temperature to 95 °C for 1 h. The rGO was recovered by filtration using cellulose filter paper and washed with 1 M HCl solution and diH_2_O to achieve neutral pH. Finally, the filtrate was vacuum-dried to obtain rGO pellets.

vi. Different weight ratios (25%, 50%, and 75%) of TiO_2_-NPs were homogenized with rGO to prepare TiO_2_@rGO-NC. Typically, TiO_2_-NPs and rGO were mixed in the presence of diH_2_O (ratio of 100 mg of solid in 2 mL of liquid) and ultrasonicated for 15 min to ensure proper homogenization of TiO_2_-NPs and rGO. One part of the as-prepared paste (TiO_2_@rGO-NC) was deposited between two Ag-electrodes to produce a sensor. The remaining part was dried at 90 °C in an oven to remove water and kept for further use in characterization studies.

vii. The sensor design comprises 3 components: two Ag-electrodes (6 cm × 0.2 cm), a glass substrate (3.2 cm × 2.4 cm) and a thin layer (≈25 μm) of sensing material (*e.g.*, TiO_2_@rGO-NC) deposited between the Ag-electrodes. The Ag-electrodes were immobilized with adhesive on the glass substrate and with the distance between the two Ag-electrodes matching the multimeter connector for resistance measurements.

viii. After deposition of the sensing material between the two Ag-electrodes, each sensor was stabilized and dried at 60 °C in an oven for 24 h. The prepared sensors were stored in a desiccator for further use.

#### Characterization of the sensor materials

X-ray diffraction (XRD) was carried out at RT using a Cu-Kα radiation source (*λ* = 1.5406 Å) at an operating voltage of 40 kV (current of 30 mA). The XRD measurements were recorded at the angle of diffraction (2*θ*) between 10° and 90°. The crystallographic parameters of the sensor materials were identified through Rietveld refinement using HighScore Plus® (v2021) connected with the ICDD® database. The XRD results were plotted using GSAS®-II (v4776) and OriginPro® (v2021).

The morphology and crystal structure of the various materials (TiO_2_-NPs, TiO_2_@rGO-NC) were evaluated by high resolution-transmission electron microscopy (HR-TEM), electron energy loss spectrometry (EELS) and selected area electron diffraction (SAED), using an FEI TECNAI F20 field emission microscope equipped with a GATAN GIF Tridiem energy filter and a GATAN Rio16 CMOS camera. For HR-TEM, the samples were prepared as follows: 5 mg TiO_2_-NPs (or TiO_2_@rGO-NC) and 5 mL diH_2_O were ultrasonicated for 20 min. Using a micropipette, 1 drop of the ultrasonicated suspension was deposited on a commercial TEM copper grid covered with a lacy carbon film. Before each measurement, the TEM sample was vacuum dried for 10 min.

Brunauer–Emmett–Teller (BET) analysis of the as-prepared materials (TiO_2_-NPs, rGO and TiO_2_@rGO NC) was carried out on a Micromeritics surface area and porosity analyzer. To determine the specific surface area (SSA), N_2_ adsorption at −196 °C was performed on an ASAP 2020 Micromeritics apparatus on 0.5 g sample, preheated under vacuum (<0.013 mbar) at 50 °C for 3 h. Evaluation of the SSA was based on the linear part of the BET analysis, and pore size distributions were obtained by applying the Barrett–Joyner–Halenda (BJH) equation to the isotherm desorption branch, and the total pore volume was estimated from the N_2_ uptake at a *P*/*P*_0_ of 0.99.

Thermogravimetry (TG), derivative thermogravimetry (DTG) and simultaneous differential thermal analysis (SDTA) of the as-prepared TiO_2_-NPs, rGO and TiO_2_@rGO NC were used to monitor the thermal properties in terms of mass change and exothermicity/endothermicity, both in air and nitrogen. The gas flow was set to 60 mL min^−1^ in the temperature range of 25–1000 °C, with a rate of 10 °C min^−1^. For each sample, 5–20 mg of the dried powder sample were measured in an alumina-150 μL sample holder.

### Room temperature methanol gas sensing and *operando* spectroscopic analysis

**Fig. 2:**
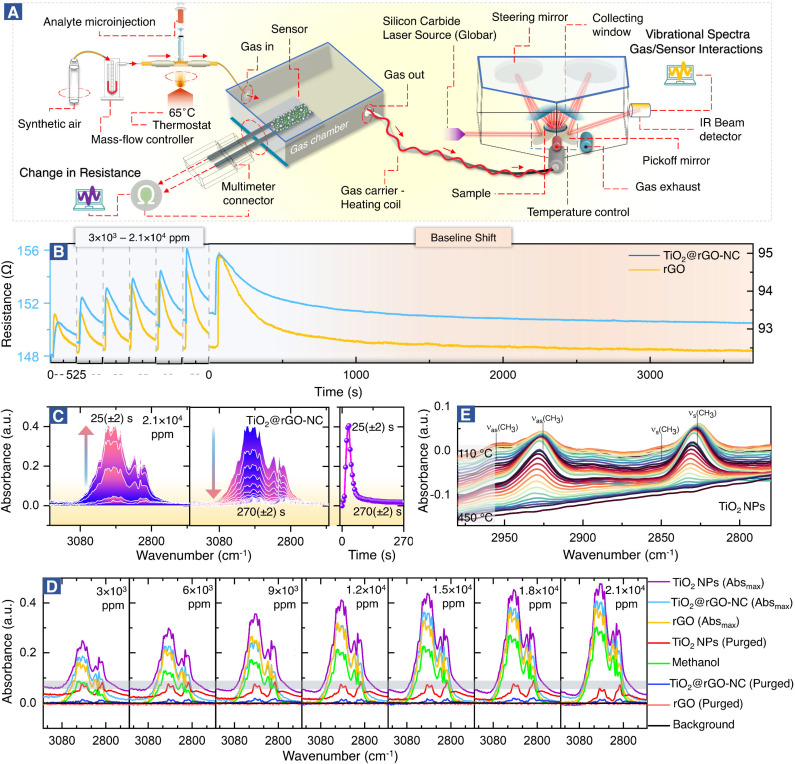
Quasi *operando* resistance/DRIFTS studies of room temperature methanol gas sensing by different sensor materials. (A) Experimental design, (B) methanol sensing and initial baseline shift, (C) time-resolved DRIFTS, adsorption–desorption studies of methanol over the 50% NC sensor, (D) *operando* DRIFTS spectra of methanol adsorption at different concentrations (3 × 10^3^ to 2.1 × 10^4^ ppm), and (E) temperature programmed desorption (TPD) of surface adsorbed methanol on TiO_2_-NPs monitored by DRIFTS.

The setup for combined room temperature methanol gas sensing and (quasi) *operando* DRIFTS, shown in Fig. 2, is described below.

### Design of the methanol gas sensing chamber

Chemiresistive methanol gas sensing measurements were performed at room temperature in an enclosed glass chamber under a continuous flow (60 mL min^−1^) of dry synthetic air (SA). Before starting each measurement, the sensor was placed inside the chamber, connected to the multimeter-connector, and allowed to stabilize under a continuous SA flow for 24 h. The desired concentration (3 × 10^3^–2.1 × 10^4^ ppm) of methanol was introduced with the steady SA flow using microinjection, with 1 μL methanol yielding 3000 ppm. For lower concentrations of 75–150 ppm, methanol was diluted with diH_2_O (ratio of 1 : 36).

The methanol molecules (gas) *vs.* chamber air molecules were calculated accordingly:Density of methanol (room temperature) = 0.7913 g mL^−1^1 μL = 0.001 mL0.001 mL × 0.7913 g mL^−1^ = 0.0007913 g = 0.7913 mg*X* = 0.7913 mg × 6.022 × 10^23^ molecules per mol/32 040 mg*X* = 1.4875 × 10^19^ moleculescalculating air molecules:*Y* = 6.022 × 10^23^ molecules per mol × 200 mL/24 460 mL*Y* = 4.9240 × 10^21^ molecules

So, the methanol molecules per air molecules are1.4875 × 10^19^/4.9240 × 10^21^ = 0.003 (3000 ppm)

The electrical resistance *R* was measured using a high-resistance multimeter, read out by UT61E software (v.4.01). The response time and recovery time of the sensor were calculated using eqn (1) and (2) which are shown in Fig. 4B,

1 Response time = *T*_max_ − *T*_start_

2 Recovery time = *T*_baseline_ − *T*_max_

Response time is defined as the time of maximum (100%) alteration in resistance in the presence of methanol (*T*_max_) minus the time at which the methanol pulse was started (*T*_start_). Recovery time is defined as the time needed by the sensor to achieve the baseline (resistance in air) (*T*_baseline_) after pulsing methanol minus the time of maximum (100%) alteration in resistance upon methanol pulsing (*T*_max_).

### *Operando*-diffuse reflectance infrared Fourier transform spectroscopy (DRIFTS) and temperature programmed desorption (TPD)

Molecular interactions of methanol gas with the sensing materials (*e.g.* rGO or TiO_2_@rGO-NC) were analyzed in real time using a downstream DRIFTS cell under the same experimental conditions as those of the resistance measurements in the environmental chamber (Fig. 1). The IR spectrometer was equipped with a silicon carbide IR source (Globar®), a liquid nitrogen-cooled mercury cadmium telluride (MCT) detector and a commercial DRIFTS mirror unit. Briefly, 50 mg of sensing material was placed in a porous ceramic sample cup and mounted in the DRIFTS cell. The spectra of the sensing material in SA served as the background and the spectra of the DRIFTS cell without the sensing material but only methanol gas (under the same SA flow) were also acquired. After a methanol gas pulse (3 × 10^3^–2.1 × 10^4^ ppm) was introduced, IR-spectra (64 scans) were recorded in 5 s intervals *via* the OPUS® (v6.5) software. For each measurement, the Abs_max_ value is defined as the maximum IR absorption for a particular methanol concentration, while the purged value is defined as the IR absorption when there is no further change over time.

Finally, for temperature-programmed methanol desorption, the sample was heated to 450 °C (10 °C min^−1^) in the DRIFTS cell while recording the IR spectra.

### *In situ* attenuated total reflectance-Fourier transform infrared (ATR-FTIR) spectroscopy

Interactions of liquid methanol with powdered sensing materials (*e.g.*, rGO, TiO_2_-NPs or TiO_2_@rGO-NC) were examined at room temperature in real time by *in situ* ATR-FTIR. The spectrometer was equipped with a liquid nitrogen-cooled mercury cadmium telluride (MCT) detector and a commercial ATR unit. The samples prepared in diH_2_O were deposited over the surface of the ZnSe crystal and air dried. The spectra of the sensing material in a steady flow of He (8 mL min^−1^) were taken as the background, while the spectra without the sensing material and with only methanol were taken as the positive control. IR spectra (64 scans) were recorded at 5 s intervals *via* the OPUS® software (v6.5) monitoring liquid methanol (1 mL) injection in a helium (He) environment inside the ATR-FTIR cell. IR spectra were recorded until there was no further signal change.

### *Operando* mass spectroscopy (MS) of room temperature methanol gas sensing

The mechanism of room temperature methanol gas sensing, previously proposed to occur *via* a surface electrochemical reaction of methanol removing charge carriers, was directly examined by *operando*-MS paralleled by resistance measurements. Thus, the methanol gas sensing chamber was connected to a quadrupole mass spectrometer (Prisma Plus QMG 220, Pfeiffer Vacuum; Fig. 5). The experimental conditions were the same as those for sensor performance measurements, but for improved detection of methanol gas and molecular species resulting from the surface electrochemical reaction, the methanol concentration was increased to 3–9 × 10^5^ ppm (injection volume = 0.1–0.3 mL). Molecular species were detected using the QMS equipped with a secondary electron multiplier (SEM) detector.

## Results and discussion

### Green synthesis and characterization of sensor materials

Fig. 1A(steps i–viii) shows the green synthesis of titanium oxide nanoparticles (TiO_2_-NPs), a nanocomposite (NC) of TiO_2_-NPs and reduced graphene oxide (TiO_2_@rGO-NC), and the sensor fabrication (for further details see the Experimental section). Olive leaves, an organic waste, are a rich source of naturally occurring metal ions chelating secondary metabolites, including secoiridoids and flavonoids. Notably, oleuropein and quercetin are highly reactive compounds and possess proven potential for reduction of metal ions to metal nanoparticles.^35,37^ In the present study, the water extract of olive leaves was used as a natural reagent to react with titanium oxysulfate in the synthesis of TiO_2_-NPs. Moreover, when the reaction conditions (*i.e.*, extract concentration, reaction temperature and pH) were compared to the classical green synthesis, it was realized that the extract of mature plant leaves was more reactive than that of young plant leaves.^34^ A possible reason may be the accumulation of nitrogenous organic compounds (NOCs) upon ageing. Also, various types of NOCs are well known to assist in producing small nanoclusters (*e.g.*, Au_10_, Au_12_ and Au_14_).^38^ In the current study, in addition to oleuropein and quercetin, NOC participation was advantageous for achieving TiO_2_-NPs of small size. Thus, using olive leaf waste in the synthesis of NPs as an alternative to synthetic reagents is not only efficient, but also represents a sustainable and environmentally friendly route. The advantages of the green synthesis method reported herein over the previously reported ones are detailed in Table S1.†

The crystallography of the as-prepared TiO_2_-NPs, rGO and a nanocomposite of TiO_2_-NPs and rGO (TiO_2_@rGO-NC) was examined by X-ray diffraction (XRD). The broad diffraction peaks (Fig. 1B) revealed the small crystallite size of the green synthesized TiO_2_-NPs. The Bragg peaks were in good agreement with ICDD 04-016-2837,^39^ indicating that TiO_2_-NPs were anatase.^28^ The crystal structure of TiO_2_-NPs was further evaluated *via* Rietveld refinement (Fig. 1B and S1†), showing unit cell parameters of *a =* 3.79 Å, *b =* 3.79 Å and *c* = 9.48 Å. The average crystallite size of TiO_2_-NPs was calculated using Debye–Scherrer's equation:

3 *D* = 0.9*λ*/*β* cos *θ*

where *D* is the average crystallite size (nm), *λ* is the X-ray wavelength (1.5406 Å), *β* is the full width at half maximum (FWHM) in radians, and *θ* is the Bragg angle. The resulting average crystallite size of TiO_2_-NPs was 4.1 nm, further detailed in Table S2.†

The XRD patterns of rGO and TiO_2_@rGO-NC are shown as an inset of Fig. 1B. The broad peak of rGO at 2*θ* = 25.27° indicates that thick carbon stacks of graphite segregated into a few layers of rGO, as commonly observed during exfoliation of graphite into graphene, graphene oxide or rGO.^40^ The results are also consistent with the powder UV-vis spectra of rGO (Note S1 and Fig. S2†). Furthermore, XRD confirmed that the structural properties of TiO_2_-NPs remained unchanged upon preparation of the nanocomposite (TiO_2_@rGO-NC).

The grain size of anatase (Fig. 1C), as determined by high-resolution transmission electron microscopy (HR-TEM) under extended Scherzer conditions, was found to be <6 nm, in good agreement with XRD. The fine-grained polycrystalline areas (Fig. 1C) were identified to be anatase by means of selected area electron diffraction (SAED; inset of Fig. 1C) and *via* the energy loss near edge structure (ELNES). The recorded ELNES, as shown in Fig. S3A,† is clearly characteristic of anatase-TiO_2_ (more details in Note S2†). The observed lattice planes are attributed to the (101) planes of anatase.

TiO_2_-NPs possess tremendous surface catalytic potential;^41,42^ however, green synthesized, homogeneous and small sized TiO_2_-NPs have been scarcely reported in the literature.^43,44^ Recently, anatase-TiO_2_ quantum dots with a crystallite size of 4.2 nm were obtained by chemical synthesis in a time-consuming and complex procedure utilizing acetylacetone, *n*-butanol, and 4-dodecylbenzene sulfonic acid.^28^ In the current study, TiO_2_-NPs with a small crystallite size (∼4 nm) and a homogeneous morphology seem to result from the synergistic effect of secondary metabolites from olive leaves, reaction temperature, and optimized pH. Maurya *et al.* described green synthesized TiO_2_ NPs of 13 nm in size of the pure anatase phase, whereas NPs prepared by a chemical synthesis method were 16 nm large.^45^ Also, Tsega *et al.* reported pure anatase-TiO_2_-NPs in a pH range between 4.4 and 6.8. A drastic change in crystallite size from 24 to 8 nm was observed when the pH was lowered to ∼3.2; but it also resulted in a loss of phase purity.^46^ A similar effect of low pH on the TiO_2_-NP phase purity was observed by Isley *et al.*^47^ Hence, a pH of ∼4 seems to be the borderline to achieve pure anatase TiO_2_-NPs.^44^

The large flakes of graphene (rGO) (Fig. S3B†) yielded a single crystalline SAED pattern (Fig. 1D), showing the typical hexagonal symmetry of (0001) orientation. The TiO_2_@rGO nanocomposite (Fig. S3B†) exhibited a homogeneous distribution of TiO_2_-NPs within the rGO matrix.

As sensor materials may need higher temperatures for operation or reactivation, the thermal properties of the prepared nanomaterials were analyzed by thermogravimetry (TG), derivative thermogravimetry (DTG) and simultaneous differential thermal analysis (SDTA) (Fig. 1E and S4†). The temperature of maximum decomposition (Tn), the relative weight loss (Δ*m*) and the temperature of 5% mass loss (defined as the decomposition temperature, Td) are summarized in Table S3† for each material. The thermal analysis of TiO_2_@rGO-NC (Fig. 1E) was distinctively different from that of TiO_2_-NPs and rGO (Fig. S4†), showing intermediate values. In air, the first weight loss until 150 °C is due to the removal of water. This endothermic process overlaps at *T* > 200 °C with the exothermic thermal decomposition of organic compounds and a maximum weight loss at *T* = 290 °C. At *T* > 400 °C, due to the presence of oxygen, a sharp oxidative decomposition of graphene occurred with an exothermic peak and a Tn of 576 °C. The presence of TiO_2_-NPs increased the thermal stability of graphene in air, increasing the temperature of maximum weight loss from *T* = 531 °C to 576 °C, while the Td increased from 290 °C to 314 °C. For *T* > 650 °C no further weight loss was recorded. This demonstrates that the TiO_2_@rGO-NC is thermally stable up to at least 300 °C and would be compatible with high temperature sensing. The thermal stability of the other sensor materials is presented in Note S3, Fig. S4 and Table S3.†

The specific surface area (SSA) of the sensing materials is an important factor for the interaction with analyte molecules and was measured using N_2_ adsorption–desorption isotherms, calculated by the Brunauer–Emmett–Teller (BET) and Barrett–Joyner–Halenda (BJH) (d*V*/d*w*) methods. The BET surface areas of TiO_2_-NPs, rGO and 50 wt% TiO_2_@rGO-NC were 101.5 m^2^ g^−1^, 36.4 m^2^ g^−1^ and 70.8 m^2^ g^−1^, respectively. Moreover, the BJH cumulative pore volume and pore width of TiO_2_-NPs, rGO and 50% TiO_2_@rGO-NC were 0.11 cm^3^ g^−1^ – 5.9 nm, 0.13 cm^3^ g^−1^ – 37.4 nm and 0.08 cm^3^ g^−1^ – 6.8 nm, respectively, as depicted in Fig. S5 (more details in Note S4†). Accordingly, upon preparation of the TiO_2_@rGO-NC the SSA was preserved. Not unexpectedly, the high SSA of TiO_2_-NPs is beneficial for MeOH sensing.^28,48,49^

### *Operando*-DRIFTS and *in situ*-ATR-FTIR spectroscopy during room temperature methanol sensing

Fig. 2A shows the setup for simultaneous room temperature measurements of the resistance *R* and the corresponding DRIFTS spectra of the sensor materials (for a pulse, the time delay between measurements is only ≤5 s). In analogy with heterogeneous catalysis, when spectroscopy is paralleled with measurements of catalytic performance, the current combined resistance/DRIFTS analysis is termed (quasi) *operando*. Fig. 2B shows how the different sensor materials respond to methanol pulses for a range of concentrations. Selected time-resolved DRIFTS spectra of methanol adsorption/desorption on TiO_2_@rGO-NC are shown in Fig. 2C (for other concentrations and rGO see Fig. S6 and S7†). The panel on the right shows the time-evolution of the peak maximum. Note that for TiO_2_@rGO-NC the response times of resistance and IR absorption coincide very well (Fig. S8†). In contrast, for rGO alone the IR response was much shorter (10–20 s) while the resistance response was delayed (35–45 s). Moreover, the recovery time differed, suggesting different bonding of the adsorbates.

Fig. 2D shows the DRIFTS spectra for various methanol concentrations and sensor materials, displaying the maximum peak intensities of each (the relative absorbance scales nearly linear with the methanol concentration; Fig. S9†), as well as those after purging. The spectra in the C–H stretching vibration range, with intensities in the order TiO_2_-NPs > TiO_2_@rGO-NC > rGO, characterize the adsorbed species. The absorption bands between 2800 and 3000 cm^−1^ result from *ν*(CH_3_) vibrations of physisorbed methanol and chemisorbed methoxy (CH_3_O). The spectra of methanol in the absence of a sensor (on the aluminum mirror; positive control) and of the background (sensor in pure synthetic air (SA); negative control) are included in Fig. 2D for reference. Interestingly, after sample purging by flowing synthetic air, all peaks disappeared for rGO, whereas some remained for TiO_2_ and TiO_2_@rGO-NC. This clearly demonstrates the important role of TiO_2_-NPs, and that most of the methanol adsorption was still reversible. To fully remove the irreversible CH_3_O species from TiO_2_*via* recombinative desorption, temperatures >400 °C are needed (Fig. 2E and S10†). This also explains the initial baseline shifts in resistance upon methanol sensing, which are attributed to irreversible methoxy adsorption modifying the sensor resistance. Fig. S10† further validates the correlation between the concentration of TiO_2_-NPs (0, 25, 50 and 75% in rGO) *versus* the initial baseline shift (sensor stability) resulting from irreversible CH_3_O adsorption on TiO_2_-NPs at room temperature. The higher the TiO_2_-NPs concentration, the higher the baseline distortion, thus affecting the sensor performance over time.

**Fig. 3:**
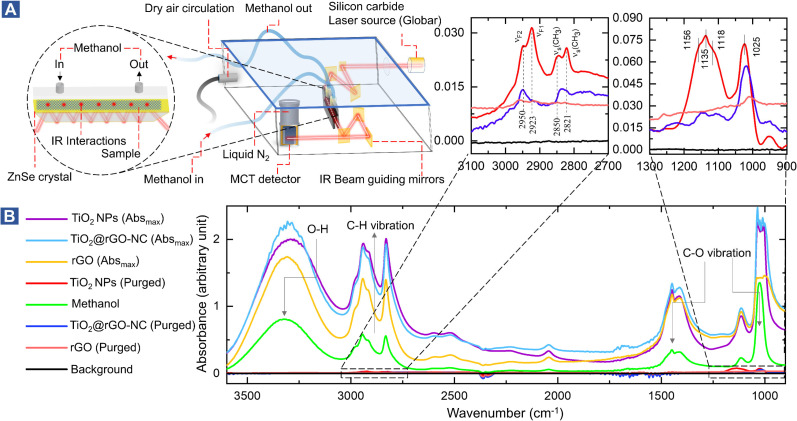
(A) Experimental design of *in situ* ATR-FTIR: vibrational spectroscopy at the liquid/solid interface (methanol on the sensor materials), (B) *in situ* ATR-FTIR spectra of 1 mL MeOH (liquid-phase) injection.

To study the interaction of the sensor materials with the analyte in more detail, liquid methanol was examined by *in situ* ATR-FTIR in the range of 1000–3600 cm^−1^, as shown in Fig. 3 (with magnified insets of selected ranges) and Fig. S11–S13.† Once more, TiO_2_-NPs enhanced the interaction, confirming the results for gaseous methanol.

After drying in He, the ATR of methanol adsorbed on TiO_2_ showed four resonances at 2821, 2850, 2923, and 2950 cm^−1^. The first two peaks are assigned to the *ν*_s_(CH_3_) mode of chemisorbed methoxy (CH_3_O) and physisorbed CH_3_OH on TiO_2_, respectively. The latter two peaks result from the Fermi resonance of chemisorbed methoxy (*ν*_F1_, CH_3_O) and physisorbed methanol (*ν*_F2_, CH_3_OH), respectively.^50–53^ Manzoli *et al.* reported that the bands between 2000 and 1000 cm^−1^ may be related to differently coordinated methoxy species on TiO_2_, with the band at 1156 cm^−1^ characterizing on-top methoxy species on Ti^3+^ near an oxygen vacancy. The band at 1135 cm^−1^ may be due to on-top species on Ti^4+^ sites.^54^ The shoulder at 1025 cm^−1^ and the peak at 1120 cm^−1^ were previously assigned to *ν*(C–O) of methanol physisorbed and chemisorbed on TiO_2_, respectively.^55^

### Sensor performance

**Fig. 4:**
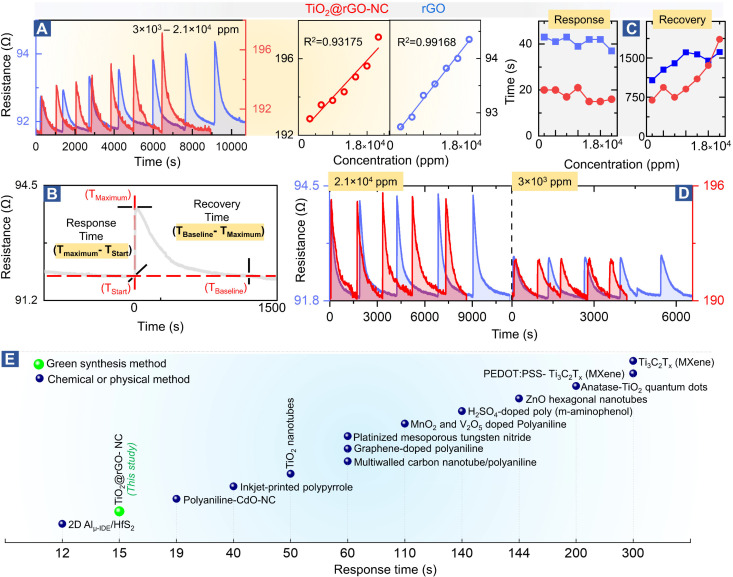
Methanol gas sensing performance of different materials: (A) room temperature methanol sensing by 50%TiO_2_@rGO-NC and rGO at different concentrations (left) and linear fit (right), (B) illustration of the sensor response and recovery time measurement, (C) sensor performance with respect to the response and recovery time, (D) reproducibility of room temperature methanol gas sensing for 50%TiO_2_@rGO and rGO at higher and ∼10-times lower methanol concentration. For even lower concentrations, see Fig. S14,† and (E) comparison of the room temperature methanol sensing response time of the current and previously reported materials (for more details, see Table S4†).

To evaluate the sensor performance in more detail (Fig. 4), each sensor was stabilized in dry synthetic air for 24 h, and then exposed to the methanol gas stimulus to establish the adsorption-induced baseline shift described above (*cf.*Fig. 2B). Once the sensor was stabilized, the room temperature methanol gas sensing performance of TiO_2_@rGO-NC and rGO (as reference) was tested for 3 × 10^3^–2.1 × 10^4^ ppm of methanol in a steady flow of SA using the chemiresistive methanol gas sensing setup (Fig. 2A).

Fig. 4A shows the relationship between the methanol gas stimulus at room temperature and the induced resistance change. It is apparent that the response increased linearly with methanol concentration. As shown in Fig. 4B, the response time of the sensor is defined in terms of time needed by the sensor to achieve 100% alteration in resistance for a particular methanol gas stimulus, while the recovery time refers to the time taken to recover from 100% to the baseline. Overall, 50% TiO_2_@rGO-NC showed the shortest response time of ∼15 s for both 1.5 × 10^4^ and 1.8 × 10^4^ ppm. Moreover, it is also clear from Fig. 4A and C that addition of TiO_2_-NPs to rGO not only shortened the sensor response time, but also the recovery time, with the latter being equally important for RT sensor operation.^27^ Apart from the linearity of resistance *vs.* methanol concentration and the response–recovery behavior, the signal reproducibility at both 3 × 10^3^ and 2.1 × 10^4^ ppm methanol was tested (Fig. 4D), with both sensing materials (TiO_2_@rGO-NC and rGO) showing excellent reproducibility. Additionally, the estimated limit of detection (LOD defined as 3 times the standard deviation SDV of the sensor in SA (baseline values without methanol gas), divided by the slope of the linear part of the calibration curve)^56^ was ∼213 and ∼847 ppm for the 50% TiO_2_@rGO-NC sensor and rGO, respectively. Moreover, the TiO_2_@rGO-NC also exhibited methanol sensitivity at much lower methanol concentrations of 75 to 150 ppm, as shown in Fig. S14.†

In light of previous studies (Fig. 4E), the current TiO_2_-NPs and the nanocomposite exhibit promising properties for methanol sensing, outperforming many previously reported materials in the response time. When synthesized by a chemical method, TiO_2_-quantum dots were able to detect 1000 ppm of methanol vapor at room temperature with a response time of ≈200 s.^28^ Similarly, Ti as a two-dimensional metal carbide (MXene) could detect 100 ppm of methanol gas at room temperature with a response time of ≈300 s.^57^ The detailed comparison in Table S4 shows the relevance of the TiO_2_@rGO-NC sensor obtained *via* a facile green synthesis, exhibiting good methanol sensitivity, short response/recovery time and reproducibility/stability, with the methanol sensing process even examined by *operando* spectroscopy herein.

### Mechanistic study of room temperature methanol sensing by *operando*-resistance/mass spectroscopy

Based on previous studies and the current *operando* and *in situ* spectroscopy of the working sensor, the mechanism of methanol gas sensing in terms of the ionosorption model^58^ can be evaluated.

### The sensor in air

The conduction channel in the sensor is through rGO, but the resistance is modified by TiO_2_, which improves the response and recovery time. Generally, TiO_2_ shows an n-type response to gas stimuli, but in the current case it exhibits a p-type response which increases the resistance upon gas exposure,^28,59^ likely due to structure defects in the nanocrystals.^60,61^ Green-synthesized TiO_2_-NPs behave like a p-type MOSc with hole carriers,^28^*i.e.*, when exposed to the dry air carrier gas, oxygen adsorbs on the surface of TiO_2_-NPs and captures electrons, forming O^−^ and O^2−^ (eqn (4)–(7)).^28^ As a result, the charge carrier density on the TiO_2_-NP surface increases by formation of a conduction layer (hole accumulation layer, HAL), providing the basis for improved sensing. In this state, the sensor surface is considered stable at room temperature in the SA carrier gas.

4 O_2(gas)_ → O_2(ads)_

5 O_2(ads)_ + e^−^ → O_2_^−^_(ads)_

6 O_2_^−^_(ads)_ + e^−^ → 2O^−^_(ads)_

7 O^−^_(ads)_ + e^−^ → O^2−^_(ads)_

### Reversible and irreversible methanol adsorption

The initial exposure of the sensor to methanol is the critical process, representing the core finding of this study. Using *operando* and *in situ* spectroscopy, it was observed that the sensing materials (TiO_2_@rGO-NC and rGO) behaved differently upon methanol gas stimulation. In the case of rGO, the first methanol exposure showed reversible adsorption and physisorbed CH_3_OH desorbed when the methanol pulse vanished upon steady air flow. As apparent from (quasi) *operando*-DRIFTS (Fig. 2D), rGO exhibited weaker methanol adsorption than the other sensing material, due to the low hydrophilicity of rGO, resulting in weak surface adsorption.^62^ This also explains the longer response time of rGO (Fig. 4C), with reversible methanol adsorption on rGO yielding a stable baseline.

The resistance of the rGO was around 100 ohms with a p-type response, and addition of TiO_2_ increased the resistance to around 200 Ω. Even more importantly, addition of TiO_2_-NPs to the rGO matrix significantly improved the sensor performance. The first methanol exposure resulted in physisorbed CH_3_OH and chemisorbed methoxy (CH_3_O), the latter being responsible for the baseline shift. Hence, reversible and irreversible RT methanol adsorption on the surface of the sensing materials controls the baseline shift and stability over time.

### Surface electrochemical reaction and interface effects

As suggested by previous reports,^58^ the interaction of methanol with TiO_2_@rGO-NC initiates a surface electrochemical reaction between CH_3_OH and the ionosorbed oxygen species (eqn (8)). This releases free electrons which neutralize the holes in TiO_2_ by electron–hole recombination (eqn (9)). Hence, the decrease in the availability of holes results in an increase in sensor resistance, as observed experimentally. Moreover, deposition of TiO_2_-NP_S_ on rGO may further accelerate methanol oxidation at the interface, leading to an increased charge carrier recombination rate, also documented by previous observations.^63^

8 CH_3_OH + 3O^2−^ → CO_2_ + 2H_2_O + 6e^−^

9 h^+^ + e^−^ → null

**Fig. 5:**
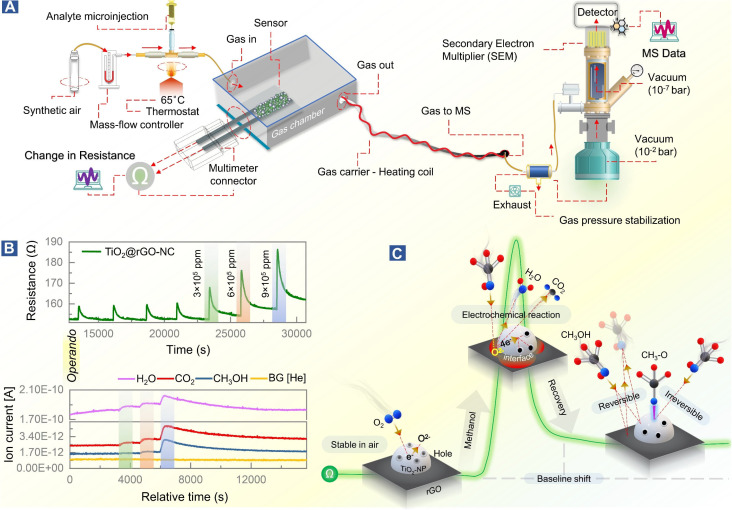
Room temperature methanol gas sensing on 50% TiO_2_@rGO-NC monitored by *operando* mass spectrometry (MS). (A) Experimental design of *operando*-resistance/mass spectroscopy of the surface electrochemical reaction upon methanol gas exposure, (B) change in resistance due to the methanol stimulus with simultaneous detection of product molecules, and (C) illustration of the methanol sensing mechanism; see text for details.

To observe this process in real time, at least in part, the methanol gas sensing chamber was connected to a mass spectrometer (MS), as shown in Fig. 5A. This allowed us to link resistance changes upon methanol stimulation with simultaneous gas phase analysis, once more in an *operando* mode (Fig. 5B). The MS detection allowed identification of products of the surface electrochemical reaction. At varying concentrations of methanol (3 × 10^5^–9 × 10^5^ ppm), H_2_O and CO_2_ were traced as products of methanol reacting with pre-adsorbed oxygen, according to eqn (8) and Fig. 5C.

Methanol conversion to CO_2_ and H_2_O due to the surface electrochemical reaction was thus evident from MS, with the tailing MS signals indicating persistent room temperature methanol oxidation. This once more highlights the importance of methanol adsorption–desorption on the surface and interfaces of TiO_2_-NPs for the resistance changes. Electron paramagnetic resonance (EPR) spectroscopy could even directly detect the formation of reactive oxygen species.^64^

However, the production of H_2_O by the surface electrochemical reaction or a high humidity at room temperature may affect the sensor performance. To address this issue, methanol gas together with vaporized H_2_O (1 : 36) was introduced to the sensing chamber. Under highly humid conditions (75 ppm methanol in 2925 ppm H_2_O), one expects a loss of functionality, but the sensor still detected up to 150 ppm methanol in 5850 ppm H_2_O before collapsing (Fig. S14†). This confirms that the sensing material is highly selective towards MeOH over H_2_O, as well as resistant towards co-dosed H_2_O.

## Conclusions

In summary, a nanocomposite methanol sensor material, consisting of TiO_2_ nanoparticles and reduced graphene oxide (rGO), was prepared by facile green chemistry routes, employing an organic olive leaf waste extract as an alternative to synthetic reagents. The TiO_2_-NPs were 4–6 nm in size and had an anatase structure. Although intended for room temperature sensing, the nanocomposite is even stable to at least 300 °C. Using a setup for (quasi) *operando*-resistance/DRIFTS measurements, the interaction of methanol with the various sensor materials was examined in real time. Methanol exposure increased the resistance, with TiO_2_@rGO-NC showing the shortest response and recovery times. The resistance scaled linearly with the methanol concentration and had a high degree of reproducibility. The room temperature molecular interactions between CH_3_OH and the sensor surface were identified as reversible and irreversible methanol adsorption, the latter due to methoxy CH_3_O formation especially on TiO_2_-NPs, leading to a resistance baseline shift. The same interactions were observed for liquid methanol by *in situ* ATR-FTIR. The mechanism of methanol gas sensing in terms of the ionosorption model was confirmed by *operando* mass spectroscopy/resistance measurements, identifying CO_2_ and H_2_O as products of a surface electrochemical reaction. Methanol adsorption releases free electrons neutralizing the holes in TiO_2_ and thus increasing the overall sensor resistance, which includes a conduction channel through rGO. The presented work may open new opportunities in *operando* monitoring of working sensor materials derived from eco- and environmentally-friendly synthesis approaches.

## Author contributions

Qaisar Maqbool: conceptualization, methodology, validation, software, formal analysis, investigation, data curation, writing – original draft, writing – review & editing. Nevzat Yigit: conceptualization, methodology, validation, formal analysis, investigation, data curation, writing – review & editing. Michael Stöger-Pollach: methodology, validation, formal analysis, investigation, writing – review & editing. Maria Letizia Ruello: validation, writing – review & editing. Francesca Tittarelli: validation, resources, writing – review & editing, supervision. Günther Rupprechter: conceptualization, validation, resources, writing – review & editing, supervision.

## Conflicts of interest

The authors declare that they have no known competing financial interests or personal relationships that could have appeared to influence the work reported in this paper.

## Supplementary Material

CY-013-D2CY01395A-s001
