# Supplementary material for: Operando monitoring of a room temperature nanocomposite methanol sensor
Source: Catal Sci Technol. 2022 Dec 14;13(3):624–36. doi: 10.1039/d2cy01395a (PMC9900598; doi:10.1039/d2cy01395a)
Supplement: CY-013-D2CY01395A-s001 [file CY-013-D2CY01395A-s001.pdf]

Electronic Supplementary Material (ESI) for Catalysis Science & Technology.
This journal is © The Royal Society of Chemistry 2022

Electronic Supplementary Information (ESI) *for*

Operando spectroscopy of a working room temperature nanocomposite methanol sensor

Qaisar Maqbool[1], Nevzat Yigit[2], Michael Stöger-Pollach[3], Maria Letizia Ruello[1], Francesca Tittarelli[1], and Günther Rupprechter[2]*

*Correspondence to: guenther.rupprechter@tuwien.ac.at

ESI includes:

Supplementary Text S1 to S4
Figs. S1 to S14
Tables S1 to S4

Supplementary Text

S1: Powder Ultraviolet-visible (UV-vis) spectroscopy of rGO

Ultraviolet-visible spectroscopy of rGO in powder phase was performed using a UV/Vis spectrometer (Perkin Elmer Lambda 750). Due to GO reduction into rGO (Fig. S2), the electronic conjugation was restored which was observed as a persistent UV absorption above 270 nm. This behavior resembles materials such as few-layered graphene nanosheets obtained via high intensity sonication of graphite [1], and highly reduced rGO [2–4]. The UV results are also consistent with the HRTEM and XRD observations, validating few-layered and highly reduced rGO.

S2: Energy Loss Near Edge Spectroscopy (ELNES) of TiO_2-NPs and HR-TEM of TiO_2@rGO-NC

Since both, rutile and anatase, have the same stoichiometry, chemical quantification via conventional scattering cross sections for the respective ionization edges cannot distinguish the two phases. On the other hand, their electronic structure differs especially for the Ti $L_{3,2}$-edge, which was recorded in detail. The energy-loss near edge structure (ELNES) reflects the density of states (DOS) above the Fermi level. The DOS itself depends on the crystallography and chemical environment of the detected chemical elements. In the case of TiO_2, the ELNES of the Ti L-$_{3,2}$-edge can be used as a fingerprint for differentiating rutile and anatase. The recorded ELNES, as shown in Fig. S3A, is clearly characteristic of anatase-TiO_2.

S3: Thermal analysis

To examine a potential use of the prepared sensor materials at higher temperatures, their thermal properties were analyzed by TG, DTG and SDTA (Fig. 1E and Fig. S4). The temperature of maximum decomposition (Tn) within one weight loss step, the relative weight loss (Δm) and the decomposition temperature (Td), i.e., the temperature of 5% weight loss, are summarized in Table S3 for each material. To evaluate the effect of oxygen in the air, corresponding studies were first performed in N_2.

The thermal behavior of TiO_2-NPs in N_2 (Fig. S4) can be divided into three stages. The first endothermic stage from room temperature to 200 °C with a mass loss of 2.6% can be attributed to the evaporation of small molecules such as physically adsorbed water (dehydration) and removal of organic solvent residues. The further weight loss from 200°C to 600°, with a global exothermic behavior, may include TiO_2 phase transformation from anatase to rutile [5,6], thermal decomposition of the peroxo titanium complex and the oxidative decomposition of carbon based compounds with maximum degradation at T = 498°C. Beyond 600° C, the endothermic peak in the DTA curve (T3 ~750°C) without weight loss reflects the phase transformation of TiO_2 from anatase to rutile [6,7]. Overall, the sample had a total of 8.8% weight loss with a Td value of 425°C.

In air, the TG curves of TiO_2-NPs showed a similar behavior, but with less evident endothermic peaks since the presence of oxygen favors exothermic processes. In this case, the first weight loss attributed to the loss of small molecular compounds was 2.5%. Then, the thermal decomposition of the titanium complex and organic residues gave rise to 2.1% and 1.4% weight loss at Tn´s of 287°C and 452°C, respectively, followed by the phase transition from anatase to rutile without significant weight loss. In total, the TiO_2-NPs lost 7.35% weight in air and Td was 404°C.

For rGO, in the presence of air (Fig. S4), the mass loss occurred in a single main step. First, up to 400°C, a slight weight decrease occurred due to the loss of VOCs and water with possibly back-to-back endo and exothermal processes. Then, at T>400°C, due to the presence of oxygen, a sharp oxidative decomposition occurred with an exothermic peak at T = 531°C and weight loss of 88%. At T>600°C, no further weight loss was recorded. The total mass loss of rGO in air was 97% and Td was 295°C.

In N_2, the weight loss of rGO due to pyrolysis was more gradual then under air. At first, VOCs and water were lost up to 150°C (T1 = 49°C). Then, thermal decomposition of organic compounds occurred which overlapped with exothermic peaks with a maximum weight loss at T = 270°C. Td was 290°C. At 1000°C, the residual weight (75%) in N_2 was higher than for RGO in air due to additional residuals of unburned char. Moreover, TG in N_2, differently from TG in air, showed a continuous weight decrease even at T >1000°C. Decomposition of rGO in N_2 primarily depended on the available functional oxygen groups. When compared to literature, the rGO of the current study was a highly reduced form of GO with least available O_2 species and stronger van der Waals forces between the layers [8] thus providing higher thermal stability and some resistance to pyrolysis at high temperature.

For thermal analysis results of TiO_2@rGO-NC in air see the main text (Fig. 1E). In brief, the TiO_2@rGO-NC was thermally stable up to at least 300°C, making it compatible with high temperature sensing. In N_2, only the Δm at T3 was significantly lower due to the absence of oxygen.

S4: BET Surface area

For rGO, the hysteresis loop appeared (Fig. S5) at relative high pressure (p/p^0), fitting best a type-III isotherm model, which reflects the hydrophobic nature of rGO as adsorbent. Such adsorption behavior is typical for adsorbate–adsorbate interaction being larger than adsorbate–sorbent interaction, e.g., water adsorption on hydrophobic surfaces (zeolites or activated carbon) [9]. On the other hand, for TiO_2-NPs and 50% TiO_2@rGO-NC the characteristic H3-hysteresis loop fits the type-IV isotherm model [10]. The BET surface area of TiO_2-NPs, rGO and 50% TiO_2@rGO-NC were 101.5 m^2/g, 36.4 m^2/g and 70.8 m^2/g, respectively. Moreover, BJH cumulative pore volume and pore width of TiO_2-NPs, rGO and 50% TiO_2@rGO-NC were 0.11 cm^3/g - 5.9 nm,0.13 cm^3/g - 37.4 nm and 0.08 cm^3/g - 6.8 nm, respectively. A high surface area, small and homogenous particle size of the green synthesized TiO_2-NPs are beneficial for their methanol gas sensing performance. Usually, metal oxide NPs with high surface area exhibit better sensitivity towards methanol sensing, which comes, however, with the limitation of higher working temperature [11]. Contrary, our study reports that TiO_2-NPs with high surface area (101.5 m^2/g) can indeed be efficiently utilized for better understanding room temperature methanol sensing.

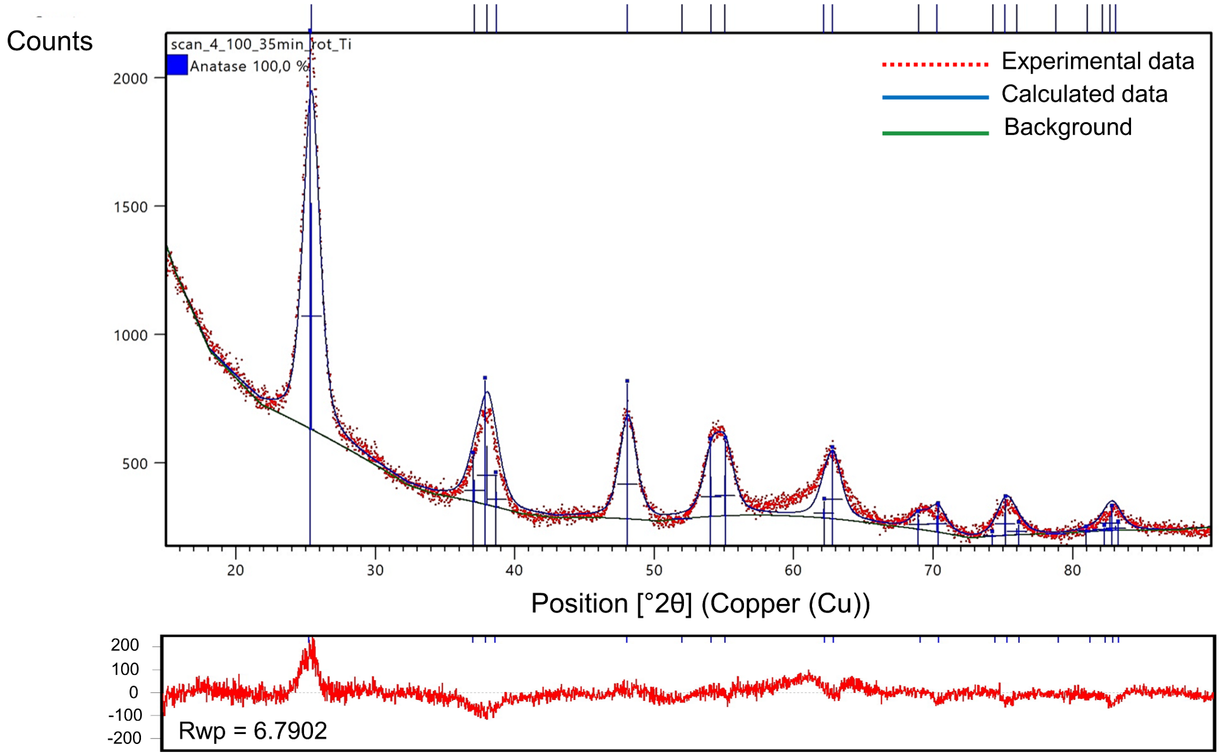

Fig S1. Rietveld refinement of TiO_2-NPs structure (XRD data) using HighScore Plus® (v2021) connected with ICDD® database (refined with ICDD 04-016-2837).

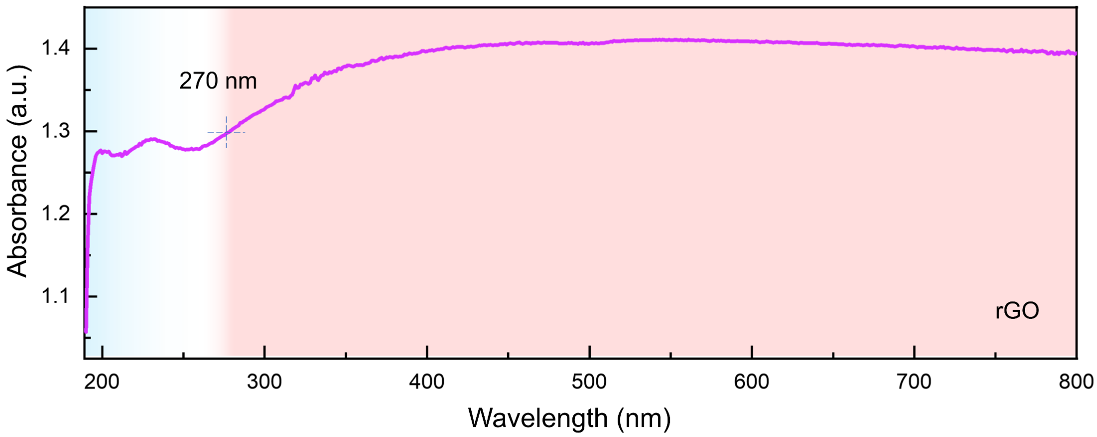

Fig. S2. UV-vis spectra (complete scan) of as synthesized rGO in powder phase showing continuous absorption above 270 nm due to reduction of GO to rGO.

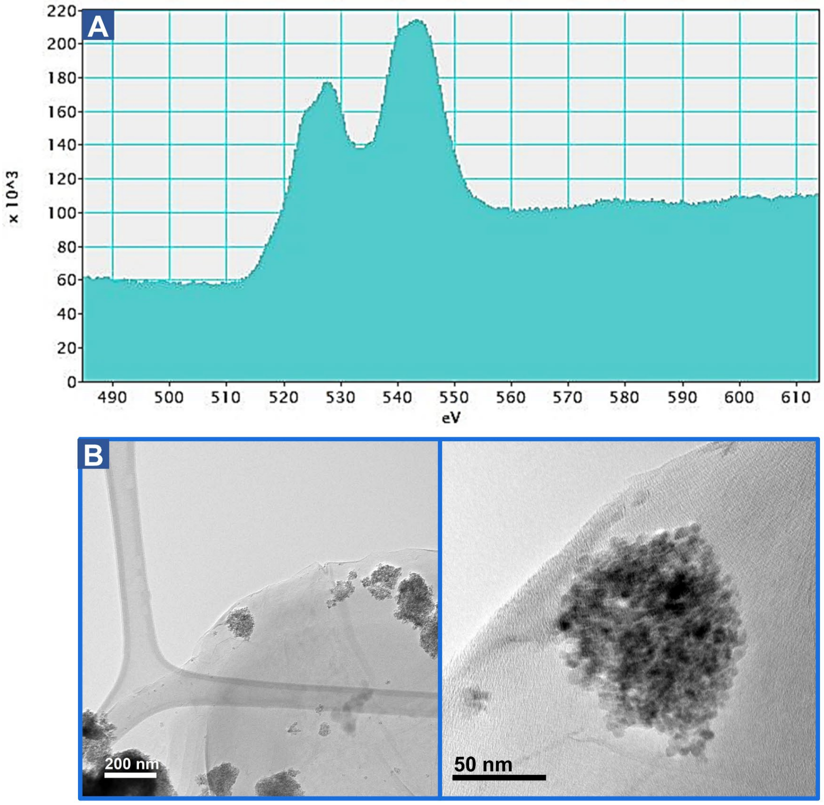

Fig. S3. (A) Energy Loss Near Edge Spectroscopy (ELNES) of synthesized Anatase-TiO_2-NPs. **(B)** HR-TEM of TiO_2@rGO-NC.

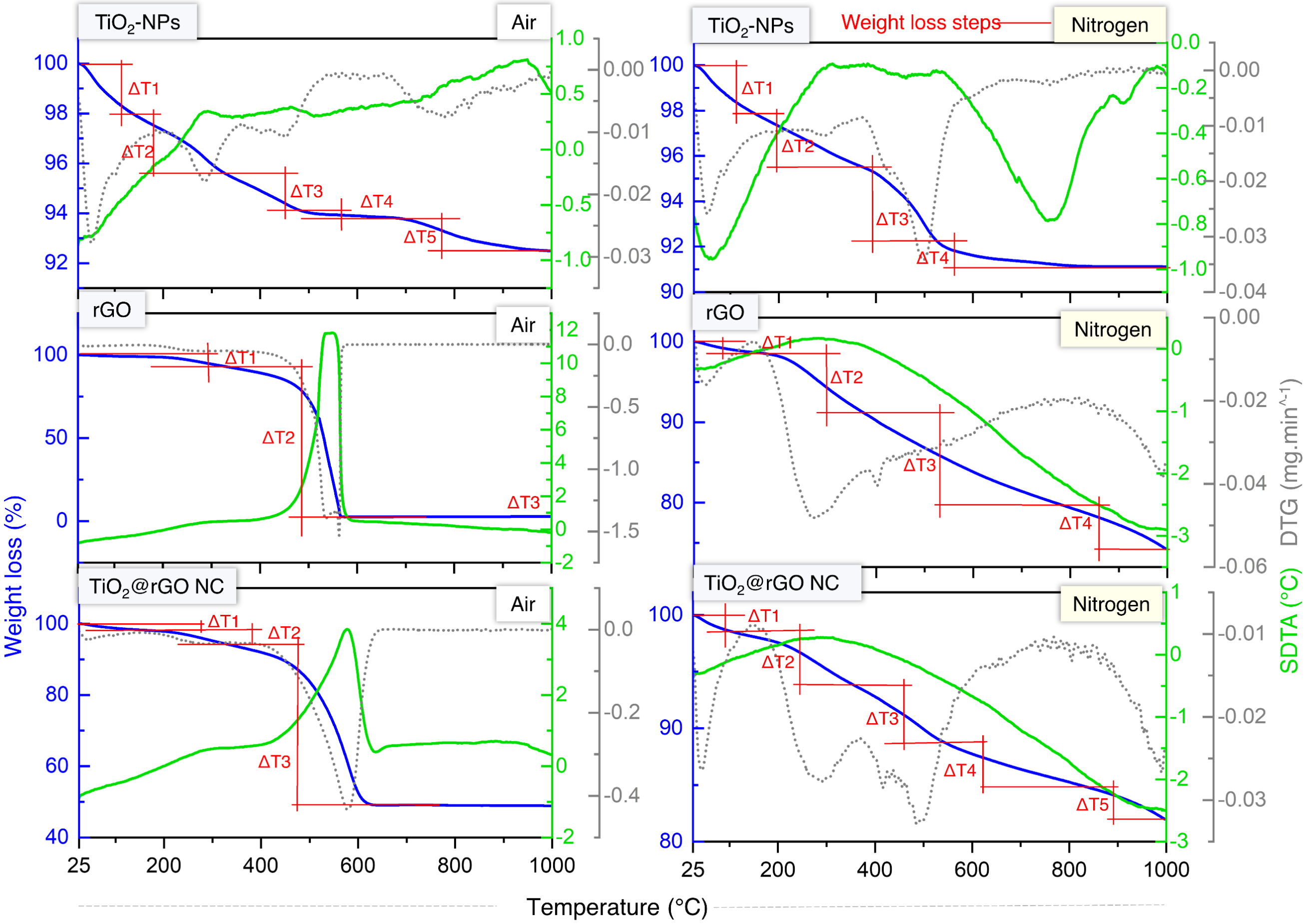

Fig. S4. TG (blue), DTG (grey) and SDTA (green) analysis of TiO_2-NPs, rGO and 50% TiO_2@rGO NC in air and nitrogen.

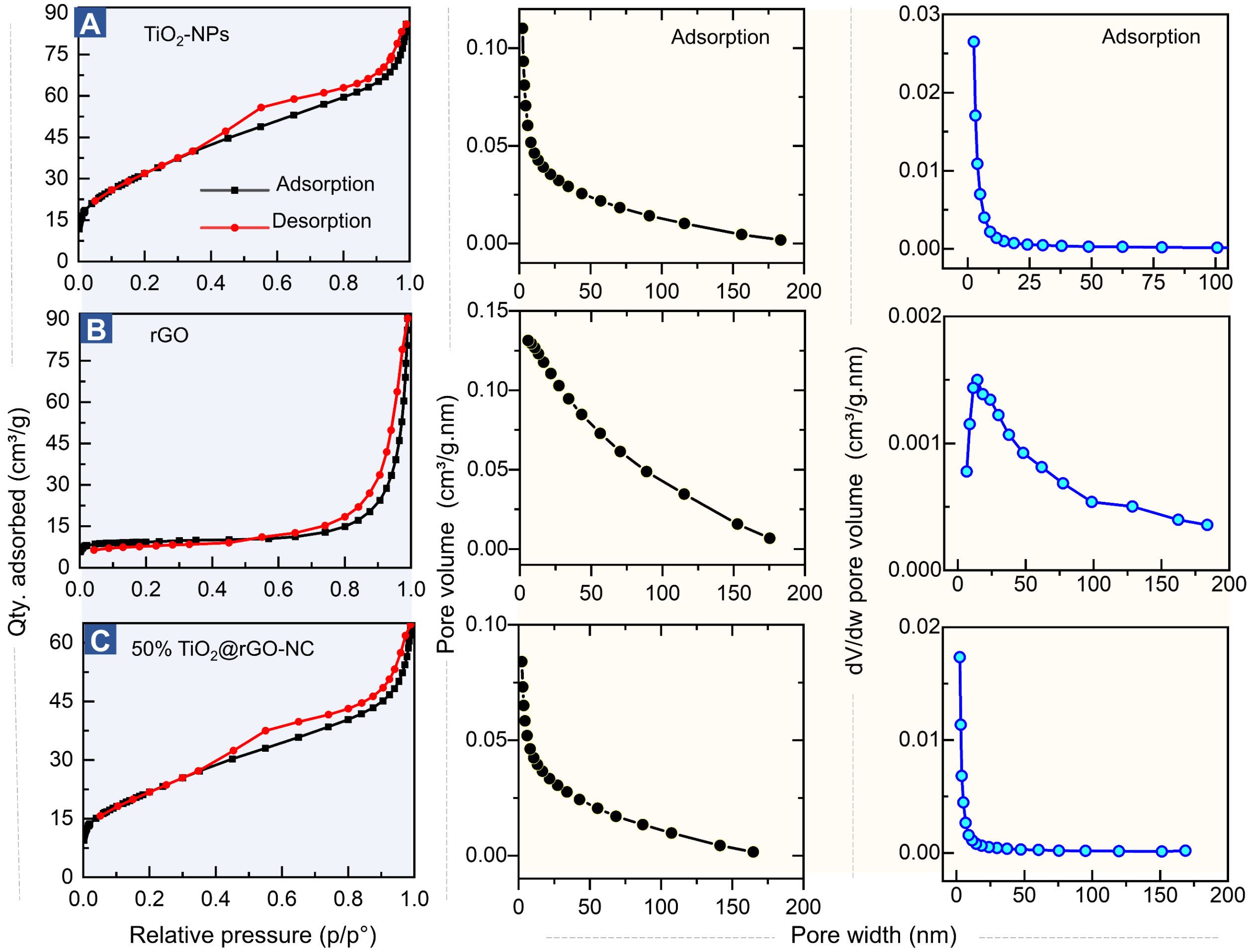

Fig. S5. BET N_2 adsorption-desorption isotherms (left) and corresponding pore size distributions (middle and right) of **(A)** green synthesized TiO_2-NPs, **(B)** rGO and **(C)** TiO_2@rGO-NC.

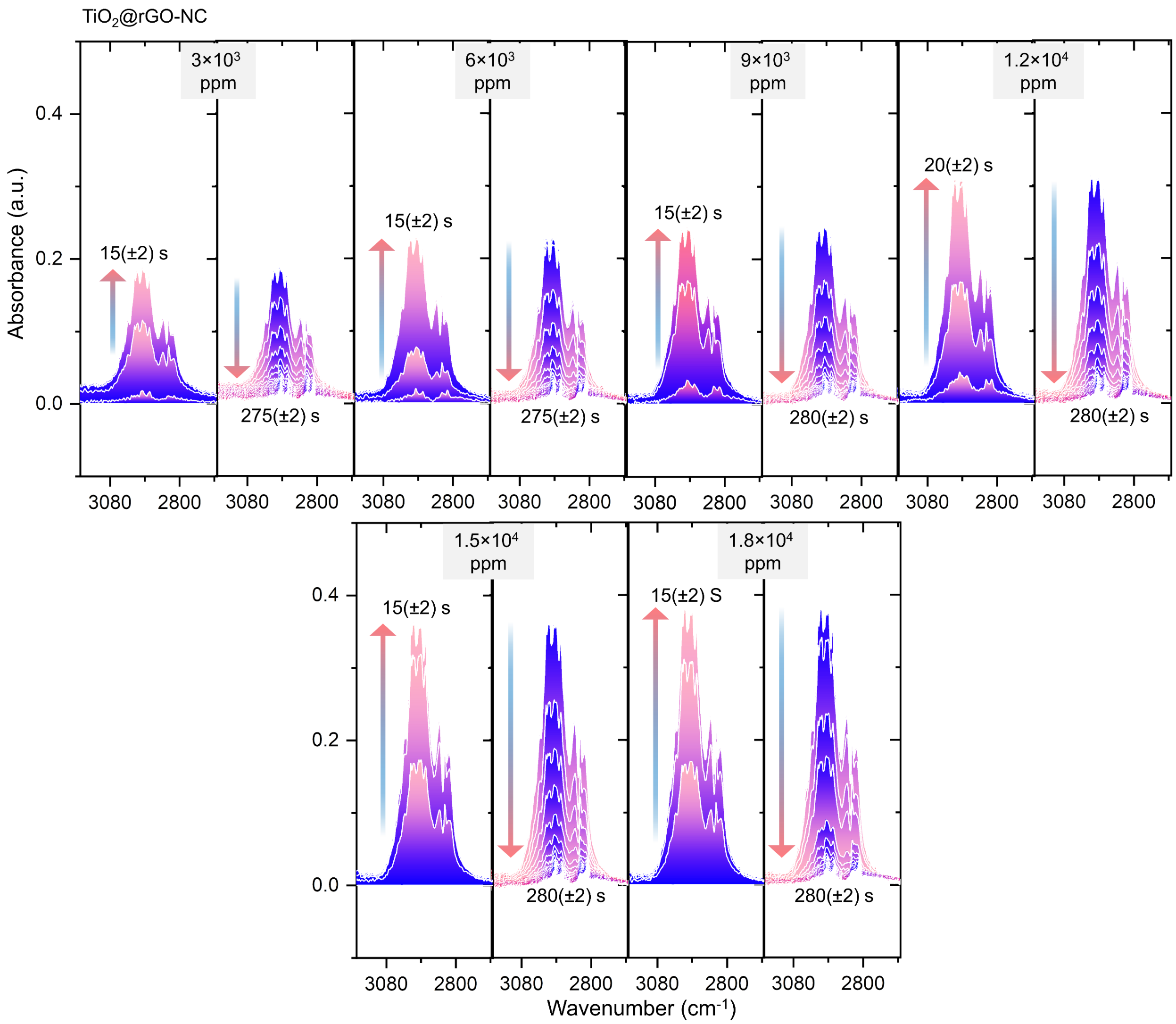

Fig. S6. Time-resolved DRIFTS of 50% TiO_2@rGO-NC, showing adsorption-desorption of methanol gas at different concentrations. The IR-derived response and recovery times are indicated.

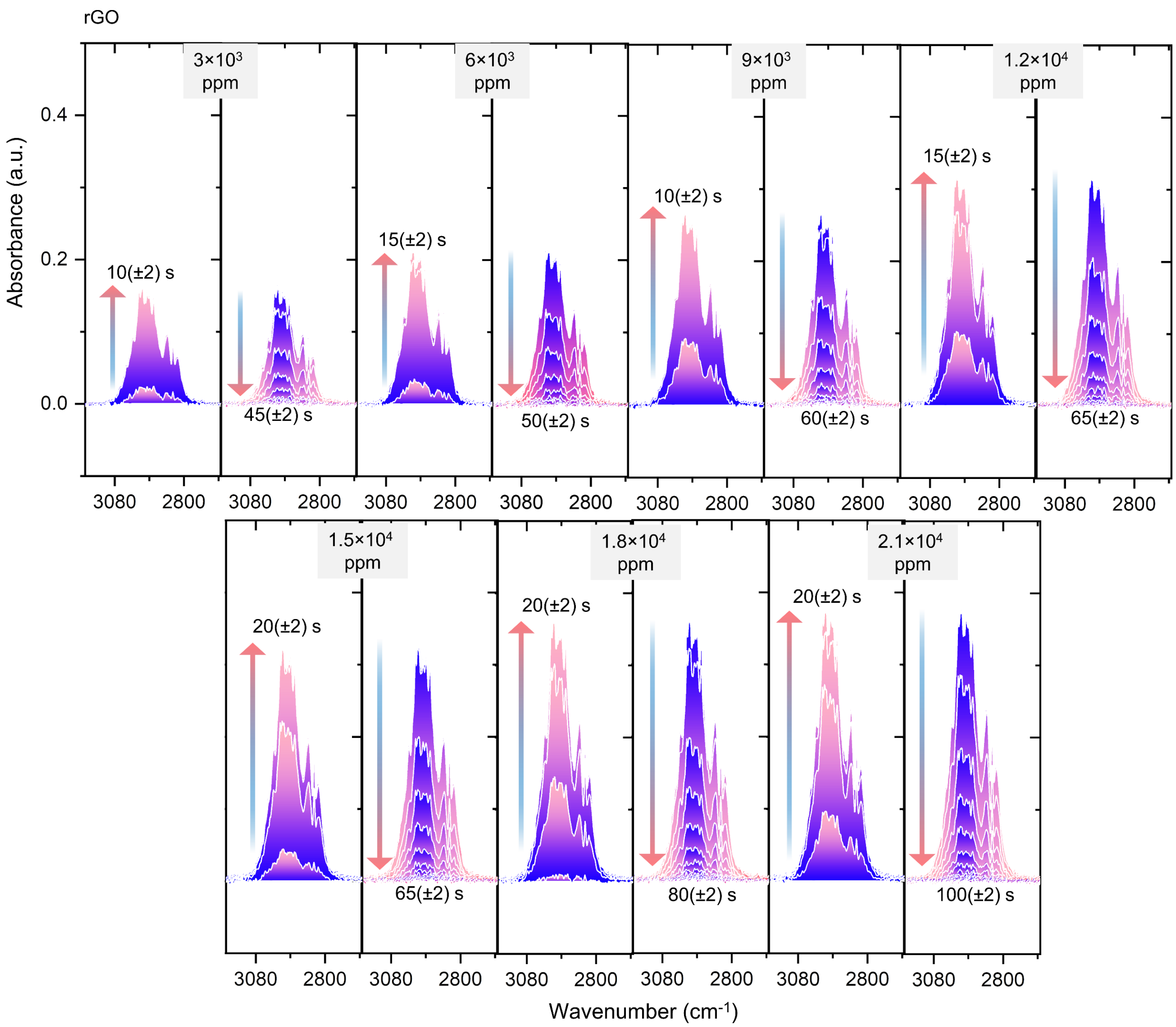

Fig. S7. Time-resolved DRIFTS of rGO, showing adsorption-desorption of methanol gas at different concentrations. The IR-derived response and recovery times are indicated.

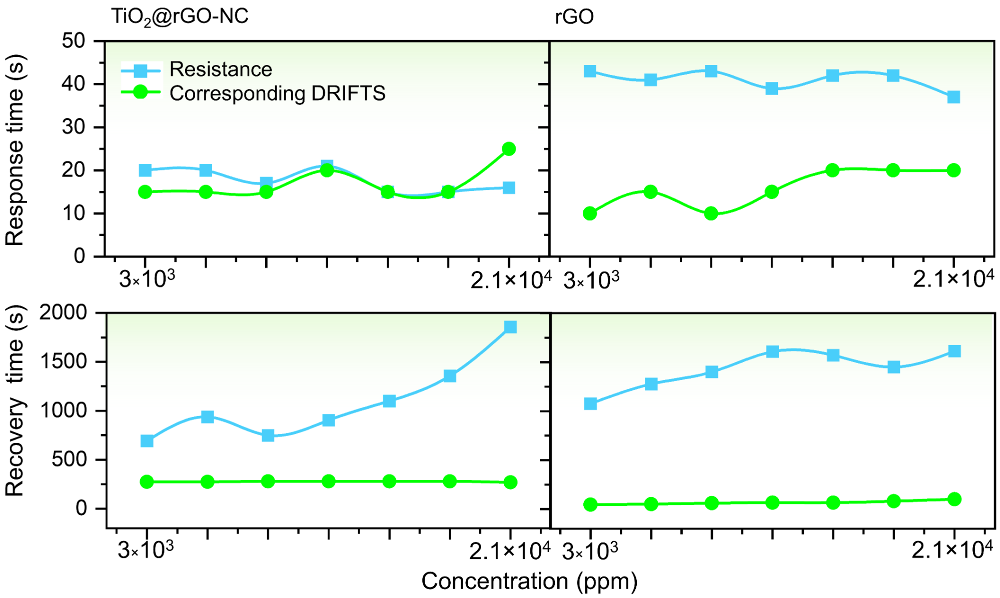

Fig. S8. Comparative analysis of sensor materials. Resistance vs. IR intensity changes, in terms of response and recovery times at different methanol concentrations (3×10^3 - 2.1×10^4 ppm).

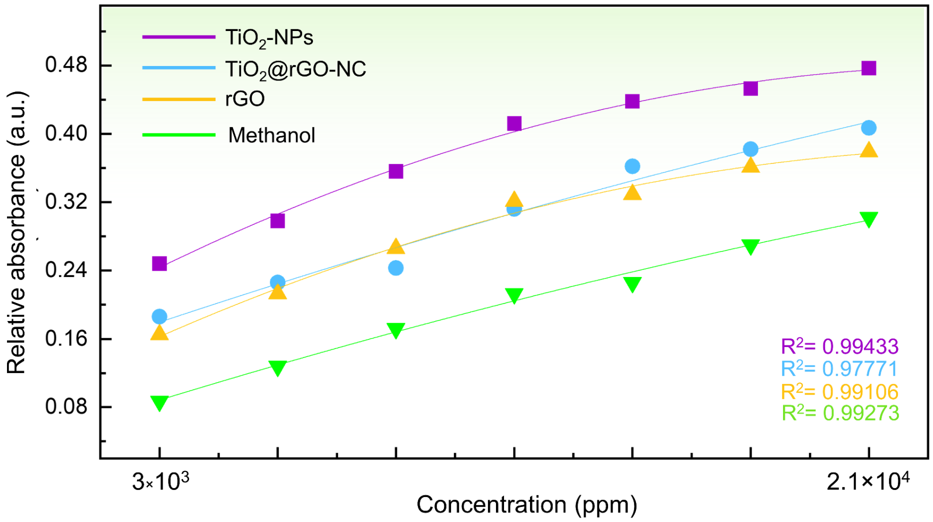

Fig. S9. Relative IR absorbance intensities for different methanol concentrations (3×10^3 - 2.1×10^4 ppm), on various sensor materials.

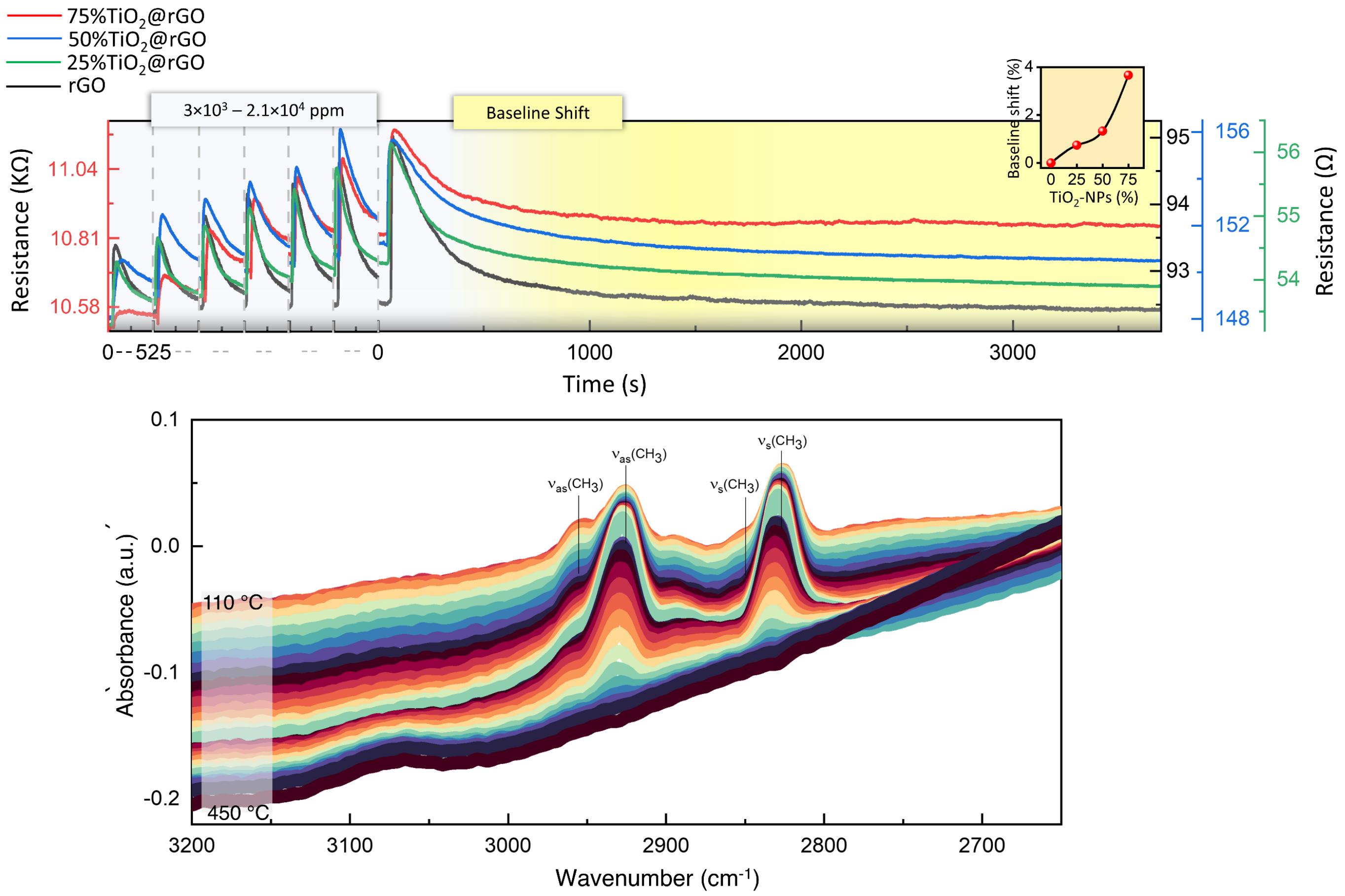

Fig. S10. Effect of TiO_2-NPs concentration (0-75%) in rGO on methanol sensing and initial baseline shift. The inset shows that the baseline shift increases for higher fraction of TiO_2-NPs. (Bottom) Temperature programmed desorption (TPD) of surface adsorbed methanol on TiO_2-NPs monitored by DRIFTS.

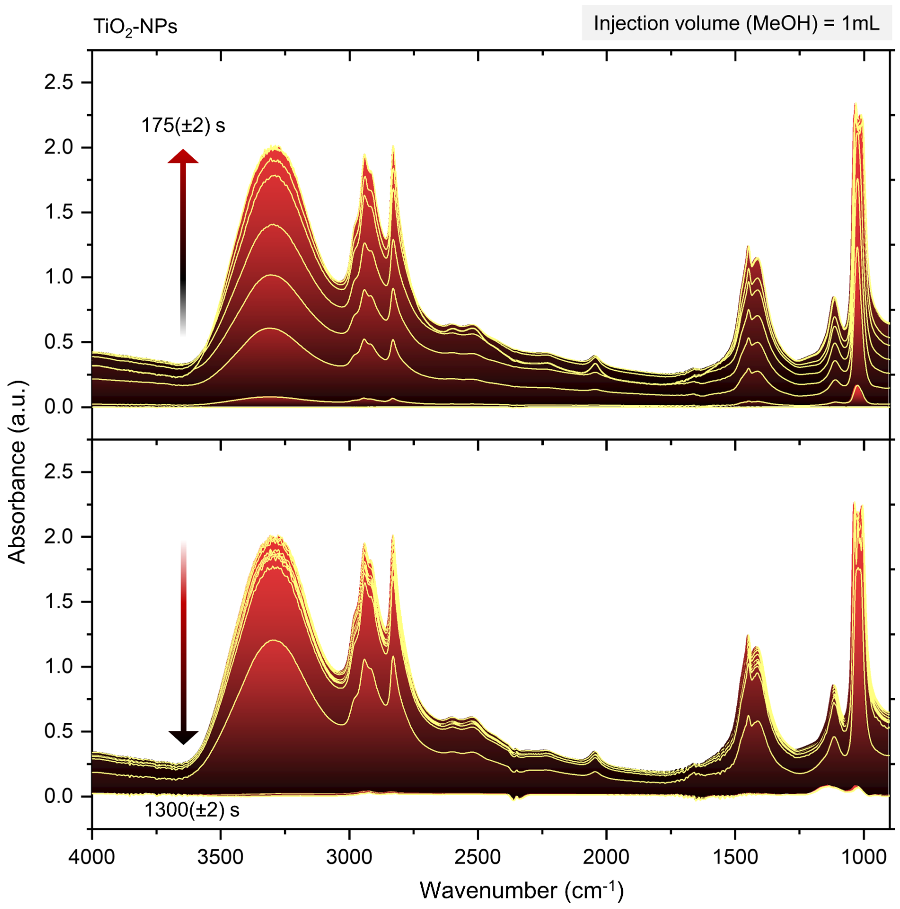

Fig. S11. Time-resolved ATR-IR of TiO_2-NPs, showing adsorption-desorption of methanol. Spectra acquired in 5 s intervals.

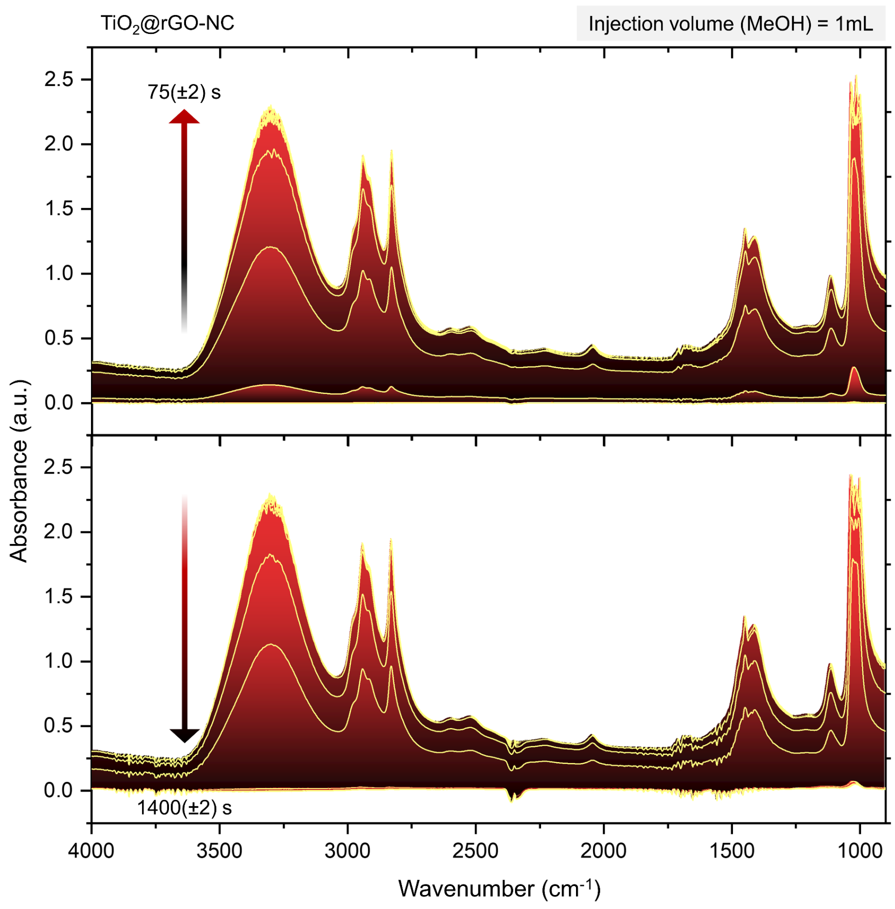

Fig. S12. Time-resolved ATR-IR of TiO_2@rGO-NC, showing adsorption-desorption of methanol. Spectra acquired in 5 s intervals.

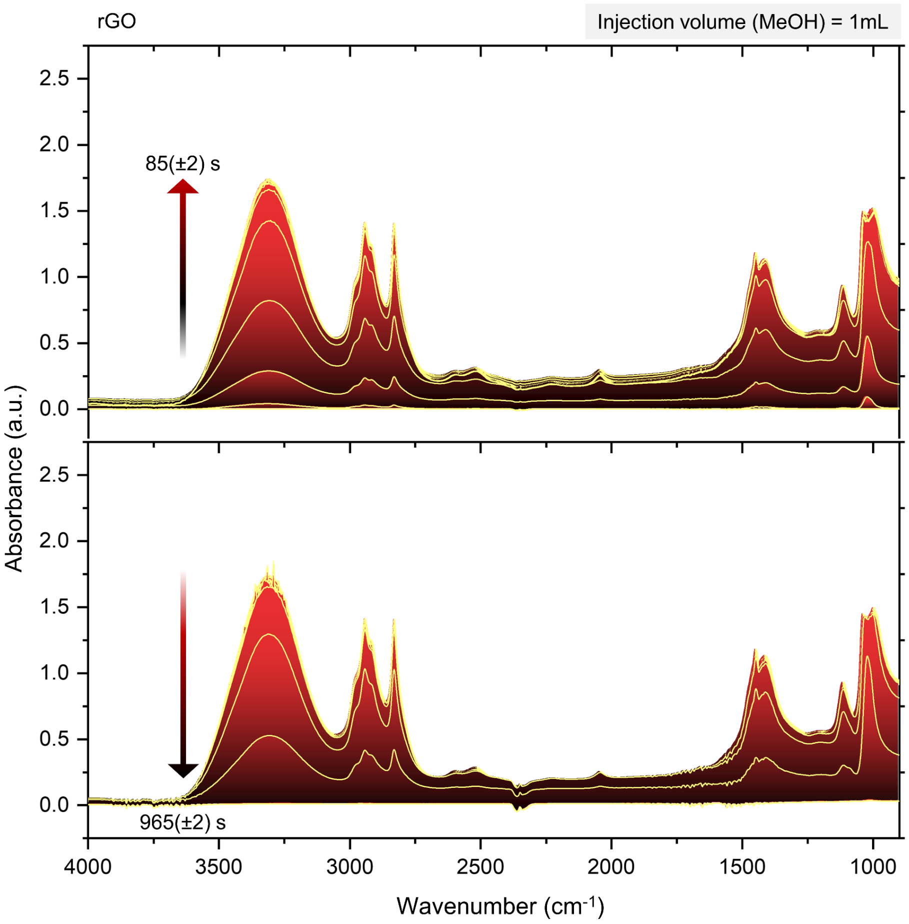

Fig. S13. Time-resolved ATR-IR of rGO, showing adsorption-desorption of methanol. Spectra acquired in 5 s intervals.

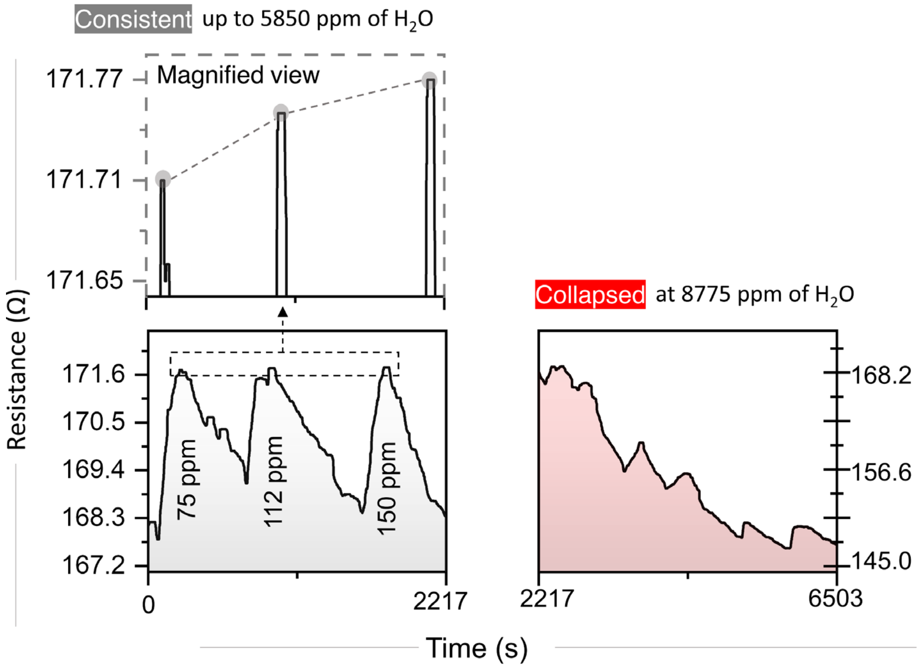

Fig. S14. Room temperature methanol detection by 50% TiO_2@rGO-NC at low methanol concentrations (75 to 150 ppm) and high humidity (2925 to 5850 ppm).

Table S1. Comparison of green synthesis methods, crystallographic and morphological properties of green synthesized TiO_2 nanoparticles.

**Biological material used**	**Precursor used**	**Particle size (nm)**	**Crystallite size (nm)**	**Morphology**	**Phase purity**	**Ref.**
Organic waste (Olive leaves)	$TiOSO_4$. xH_2SO_4	≤ 6	4.1	Spherical	Anatase	This work
Syzygium cumini (Fresh leaves)	$Ti[OCH(CH_3)_2]_4$	18	10	Spherical/irregular	Anatase	[12]
Withania somnifera (Fresh roots)	TiO_2	40-100	45.3	Spherical/porous	Anatase	[13]
Citrus limetta (Fresh leaves)	$Ti(OBu)_4$	80-100	45	Spherical	Anatase	[14]
Phyllanthus niruri (Fresh leaves)	$Ti[OCH(CH_3)_2]_4$	≥ 100	20.9	Irregular	Anatase	[15]
Calotropis gigantea (Fresh leaves)	$TiCl_4$	13-110	11.9	Flower-like	Rutile	[16]
Mangifera indica L. (Fresh fruit peel)	$TiCl_3$	17-28	5.3-13.8	Spherical/granular	Anatase-Rutile	[17]
Pouteria campechiana (Fresh leaves)	TiO_2	73-140	29	Polydisperse	Anatase	[18]
Ledebouria revoluta (Fresh bulbs)	TiO_2	100-200	47	Spherical	Rutile	[19]
Cuminum cyminum (Seeds)	$Ti[OCH(CH_3)_2]_4$	12.6	15.2	Spherical/irregular	Anatase	[20]
Monsonia burkeana (Fresh plant)	TiF_4	6-10	8.9	Spherical	Anatase	[21]
Catharanthus roseus (Fresh leaves)	TiO_2	25-110	100	Irregular	Anatase	[22]
Trigonella foenum (Fresh leaves)	$TiOSO_4$. xH_2SO_4	20-90	25-30	Spherical	Anatase	[23]
Piper betel (Dried leaves)	$Ti(OBu)_4$	8	4.0	Spherical	Rutile	[24]
Psidium guajava (Fresh leaves)	$TiO(OH)_2$	32.6	NA[1]	Spherical	Rutile/Anatase	[25]

Annona squamosa (Fruit peel)	$TiO(OH)_2$	23	NA[1]	Spherical/polydisperse	Rutile	26
Jatropha curcas L. (Crude latex)	$TiO(OH)_2$	25-100	NA[1]	Spherical/irregular	Anatase	27
Euphorbia prostrata (Fresh leaves)	$TiO(OH)_2$	83.2	NA[1]	Polydisperse	Anatase	28
Sargassum myriocystum (Fresh seaweed)	TiO_2	~50–90	NA[1]	Irregular	Anatase	29
Eclipta prostrata (Fresh leaves)	$TiO(OH)_2$	36-68	NA[1]	Spherical/polydisperse	Rutile	30
Azadirachta indica (Fresh leaves)	$Ti[OCH(CH_3)_2]_4$	124	NA[1]	Spherical	NA[1]	31

[1] NA= Not available (information not found)

Table S2. Crystallographic parameters of green synthesized anatase-TiO_2-NPs.

Peak pos. [°2Theta]		*hkl*	FWHM[1]		Crystallite size[2] [nm]		Average crystallite size [nm]		d- Spacing [nm]	
**Raw data**	**Baseline corr.**		**Raw data**	**Baseline corr.**	**Raw data**	**Baseline corr.**	**Raw data**	**Baseline corr.**	**Raw data**	**Baseline corr.**
25.40	25.42	101	1.49	1.60	5.45	5.08	3.97	4.14	0.350	0.349
37.91	37.95	004	2.29	1.98	3.65	4.22			0.237	0.236
48.11	48.11	200	1.55	1.63	5.60	5.33			0.188	0.188
54.66	54.61	105	2.86	2.83	3.11	3.15			0.167	0.167
62.62	62.72	204	2.82	2.16	3.29	4.28			0.148	0.148
69.30	69.66	116	6.21	4.34	1.55	2.22			0.135	0.134
75.36	75.36	215	2.25	2.31	4.45	4.33			0.126	0.126
83.03	83.08	224	2.29	2.34	4.63	4.53			0.116	0.116

[1]FWHM were measured using Lorentz peak fitting for both raw data and after baseline correction using OriginPro® (V2021).
[2]Crystallite size were calculated using Scherrer equation

Table S3. Thermal properties for different sensor materials. The temperatures of maximum decomposition (Tn), the relative weight loss (Δm) and the temperature of 5% mass loss, defined as decomposition temperature (Td), are summarized for each material.

**Sample**	**Properties**	**Air**					**Nitrogen**				
		**T1**	**T2**	**T3**	**Td**	**Total Δm %**	**T1**	**T2**	**T3**	**Td**	**Total Δm %**
TiO_2-NPs	Tn (°C)	54	287	452	404	7.3	53	294	498	425	8.8
	- Δm (%)	2.5	2.1	1.4	5		2.6	1.7	3.9	5.0	
rGO	Tn (°C)	531			295	97.0	49	270		290	24.9
	- Δm (%)	88.0			5		1.1	19.0		5	
50% TiO_2@rGO NC	Tn (°C)	44	290	576	314	51.0	43	292	488	319	17.8
	- Δm (%)	1.6	6.3	42.9	5		1.6	4.8	7.6	5.0	

Table S4. Comparison of the room temperature methanol sensing performance and mechanism of the prepared sensors with previously reported materials.

Sensing Material Description	Synthesis Method	Description of Sensing Mechanism	Response Time (s)	Detection Range (ppm)	Reversibility Test	Ref.
TiO_2@reduced graphene oxide nanocomposite	Green synthesis	*Operando* and *in situ* spectroscopy	15 (100%)	$75 – 9×10^5$	Tested	This study
TiO_2 nanotubes	Anodic oxidation process	Wolkenstein adsorption-desorption theory	≈50 (100%)	100 - 300	N/A	[32]
Graphene-doped polyaniline	Chemical oxidation	Theoretical fitting using Langmuir kinematic	60 (100%)	50 - 100	Tested	[33]
2D $Al_{\mu\text{-IDE}}/HfS_2$	Chemical	Langmuir adsorption-desorption isotherm	~12 (67%)	100-500	N/A	[34]
ZnO hexagonal nanotubes	Electrodeposition / Electrochemical etching	Langmuir-Hinshelwood isotherm	144 (90%)	10-700	Tested	[35]
Multi-walled carbon nanotube/ polyaniline nanotube	Chemical	Density functional theory	60 (100%)	50 - 500	Tested	[36]
Anatase TiO_2 quantum dots	Chemical	Comparing Inert gas (N_2) with O_2 environment	≈200 (100%)	1000	Tested	[37]
$Ti_3C_2T_x$ (MXene)	Chemical	Hypothetical	≈300 (100%)	100	Tested	[38]
Inkjet-printed polypyrrole	Physio-Chemical (polypyrrole over polyester)	Hypothetical	40 (90%)	5000	Tested	[39]
Platinized mesoporous tungsten nitride	Solid state reaction	Hypothetical	60 (90%)	25-200	Tested	[40]
Polyaniline-CdO nanocomposite	Chemical/ Co-precipitation / dip-coating	Hypothetical	19 (90%)	100	N/A	[41]
MnO_2 and V_2O_5 doped Polyaniline	Chemical	Hypothetical	110 (100%)	40 - 60	N/A	[42]
Carbon derivatives in poly(etherimide)–liquid crystal polymer composite	Chemical	Hypothetical	N/A	300-1200	Tested	[43]
H_2SO_4 doped poly (m-aminophenol)	Chemical	N/A	≈140 (100%)	173 - 330	Tested	[44]

PEDOT:PSS and $Ti_3C_2T_x$ (4:1)	Chemical	N/A	≈300 (100%)	300	N/A	[45]
In_2O_3 over Au, Ag, Pt and Cu	Vapor deposition	N/A	N/A	200 - 900	N/A	[46]

ppm = Part per million, N/A = Not available

Supplementary References

1 M. Noroozi, A. Zakaria, S. Radiman and Z. A. Wahab, *PLoS One*, 2016, **11**, e0152699.
2 B. A. Aragaw, *J. Nanostructure Chem. 2019 101*, 2019, **10**, 9–18.
3 S. Yang, W. Yue, D. Huang, C. Chen, H. Lin and X. Yang, *RSC Adv.*, 2012, **2**, 8827–8832.
4 C. P. P. Wong, C. W. Lai, K. M. Lee and S. B. Abd Hamid, *Mater. 2015, Vol. 8, Pages 7118-7128*, 2015, **8**, 7118–7128.
5 E. R. Spada, E. A. Pereira, M. A. Montanhera, L. H. Morais, R. G. Freitas, R. G. F. Costa, G. B. Soares, C. Ribeiro and F. R. de Paula, *J. Mater. Sci. Mater. Electron.*, 2017, **28**, 16932–16938.
6 X. Zhu, S. Han, W. Feng, Q. Kong, Z. Dong, C. Wang, J. Lei and Q. Yi, *RSC Adv.*, 2018, **8**, 14249–14257.
7 M. M. Mahlambi, A. K. Mishra, S. B. Mishra, R. W. Krause, B. B. Mamba and A. M. Raichur, *J. Therm. Anal. Calorim.*, 2012, **110**, 847–855.
8 T. Tene, G. T. Usca, M. Guevara, R. Molina, F. Veltri, M. Arias, L. S. Caputi and C. V. Gomez, *Nanomater. 2020, Vol. 10, Page 279*, 2020, **10**, 279.
9 M. A. Al-Ghouti and D. A. Da'ana, *J. Hazard. Mater.*, 2020, **393**, 122383.
10 S. Subudhi, G. Swain, S. P. Tripathy and K. Parida, *Inorg. Chem.*, 2020, **59**, 9824–9837.
11 Q. Rong, Y. Zhang, J. Hu, K. Li, H. Wang, M. Chen, T. Lv, Z. Zhu, J. Zhang and Q. Liu, *Sci. Reports 2018 81*, 2018, **8**, 1–12.
12 N. K. Sethy, Z. Arif, P. K. Mishra and P. Kumar, *Green Process. Synth.*, 2020, **9**, 171–181.
13 N. A. Al-Shabib, F. M. Husain, F. A. Qais, N. Ahmad, A. Khan, A. A. Alyousef, M. Arshad, S. Noor, J. M. Khan, P. Alam, T. H. Albalawi and S. A. Shahzad, *Front. Microbiol.*, 2020, **11**, 1680.
14 G. Nabi, A. Majid, A. Riaz, T. Alharbi, M. Arshad Kamran and M. Al-Habardi, *Inorg. Chem. Commun.*, 2021, **129**, 108618.
15 A. Panneerselvam, J. Velayutham and S. Ramasamy, *IET Nanobiotechnology*, 2021, **15**, 164–172.
16 P. Naresh Kumar Reddy, D. P. M. D. Shaik, V. Ganesh, D. Nagamalleswari, K. Thyagarajan and P. Vishnu Prasanth, *Appl. Surf. Sci.*, 2021, **561**, 150092.
17 I. N. Isnaeni, Indriyati, Dedi, D. Sumiarsa and I. Primadona, *Mater. Lett.*, 2021, **294**, 129792.
18 M. Narayanan, P. G. Devi, D. Natarajan, S. Kandasamy, K. Devarayan, M. Alsehli, A. Elfasakhany and A. Pugazhendhi, *Environ. Res.*, 2021, **200**, 111333.
19 R. Aswini, S. Murugesan and K. Kannan, *https://doi.org/10.1080/03067319.2020.1718668*, 2020, **101**, 2926–2936.
20 S. S. Mathew, N. E. Sunny and V. Shanmugam, *Inorg. Chem. Commun.*, 2021, **126**, 108485.
21 N. M. Ngoepe, M. M. Mathipa and N. C. Hintsho-Mbita, *Optik (Stuttg).*, 2020, **224**, 165728.
22 K. Velayutham, A. A. Rahuman, G. Rajakumar, T. Santhoshkumar, S. Marimuthu, C. Jayaseelan, A. Bagavan, A. V. Kirthi, C. Kamaraj, A. A. Zahir and G. Elango, *Parasitol. Res.*, 2012, **111**, 2329–2337.
23 S. Subhapriya and P. Gomathipriya, *Microb. Pathog.*, 2018, **116**, 215–220.

24 S. M. Hunagund, V. R. Desai, J. S. Kadadevarmath, D. A. Barretto, S. Vootla and A. H. Sidarai, *RSC Adv.*, 2016, **6**, 97438–97444.
25 T. Santhoshkumar, A. A. Rahuman, C. Jayaseelan, G. Rajakumar, S. Marimuthu, A. V. Kirthi, K. Velayutham, J. Thomas, J. Venkatesan and S. K. Kim, *Asian Pac. J. Trop. Med.*, 2014, **7**, 968–976.
26 S. M. Roopan, A. Bharathi, A. Prabhakarn, A. Abdul Rahuman, K. Velayutham, G. Rajakumar, R. D. Padmaja, M. Lekshmi and G. Madhumitha, *Spectrochim. Acta Part A Mol. Biomol. Spectrosc.*, 2012, **98**, 86–90.
27 M. Hudlikar, S. Joglekar, M. Dhaygude and K. Kodam, *Mater. Lett.*, 2012, **75**, 196–199.
28 A. A. Zahir, I. S. Chauhan, A. Bagavan, C. Kamaraj, G. Elango, J. Shankar, N. Arjaria, S. M. Roopan, A. A. Rahuman and N. Singh, *Antimicrob. Agents Chemother.*, 2015, **59**, 4782–4799.
29 P. Balaraman, B. Balasubramanian, W. C. Liu, D. Kaliannan, M. Durai, H. Kamyab, M. Alwetaishi, V. Maluventhen, V. Ashokkumar, S. Chelliapan and A. Maruthupandian, *Environ. Res.*, 2022, **204**, 112278.
30 G. Rajakumar, A. A. Rahuman, B. Priyamvada, V. G. Khanna, D. K. Kumar and P. J. Sujin, *Mater. Lett.*, 2012, **68**, 115–117.
31 R. Sankar, K. Rizwana, K. S. Shivashangari and V. Ravikumar, *Appl. Nanosci.*, 2015, **5**, 731–736.
32 P. Bindra and A. Hazra, *Sensors Actuators B Chem.*, 2019, **290**, 684–690.
33 R. Pal, S. L. Goyal, I. Rawal and S. Sharma, *Iran. Polym. J. (English Ed.*, 2020, **29**, 591–603.
34 S. Das, S. Sharma and S. K. Sharma, *IEEE Sens. J.*, 2019, **19**, 9090–9096.
35 D. Acharyya and P. Bhattacharyya, *Sensors Actuators B Chem.*, 2016, **228**, 373–386.
36 A. Bora, K. Mohan, D. Pegu, C. B. Gohain and S. K. Dolui, *Sensors Actuators B Chem.*, 2017, **253**, 977–986.
37 A. Šutka, R. Eglitis, A. Kuzma, K. Smits, A. Zukuls, J. D. Prades and C. Fàbrega, *ACS Appl. Nano Mater.*, 2021, **4**, 2522–2527.
38 E. Lee, A. Vahidmohammadi, B. C. Prorok, Y. S. Yoon, M. Beidaghi and D. J. Kim, *ACS Appl. Mater. Interfaces*, 2017, **9**, 37184–37190.
39 M. F. Mabrook, C. Pearson and M. C. Petty, *Sensors Actuators B Chem.*, 2006, **115**, 547–551.
40 D. Meng, S. Zhang, T. Thomas, C. Huang, J. Zhao, R. Zhao, Y. Shi, F. Qu and M. Yang, *Sensors Actuators B Chem.*, 2020, **307**, 127686.
41 R. Paulraj, P. Shankar, G. K. Mani, L. Nallathambi and J. B. B. Rayappan, *J. Electron. Mater. 2018 4710*, 2018, **47**, 6000–6006.
42 R. Pal, S. L. Goyal and S. Sharma, *AIP Conf. Proc.*, 2019, **2115**, 030217.
43 S. Bag, K. Rathi and K. Pal, *Nanotechnology*, 2017, **28**, 205501.
44 P. Kar, N. C. Pradhan and B. Adhikari, *Sensors Actuators B Chem.*, 2009, **140**, 525–531.
45 X. Wang, K. Sun, K. Li, X. Li and Y. Gogotsi, *Chinese Chem. Lett.*, 2020, **31**, 1018–1021.
46 N. G. Patel, P. D. Patel and V. S. Vaishnav, *Sensors Actuators B Chem.*, 2003, **96**, 180–189.
